# Multi-gene risk score for prediction of clinical outcomes in treatment-naïve metastatic castrate-resistant prostate cancer

**DOI:** 10.1093/jncics/pkaf025

**Published:** 2025-02-28

**Authors:** Muhammad Zaki Hidayatullah Fadlullah, David Nix, Cameron Herberts, Corinne Maurice-Dror, Alexander W Wyatt, Bogdana Schmidt, Brayden Fairbourn, Aik-Choon Tan, Liang Wang, Manish Kohli

**Affiliations:** Department of Oncological Sciences, Huntsman Cancer Institute, University of Utah, Salt Lake City, UT, United States; Department of Biomedical Informatics, Huntsman Cancer Institute, University of Utah, Salt Lake City, UT, United States; Department of Biomedical Informatics, Huntsman Cancer Institute, University of Utah, Salt Lake City, UT, United States; Vancouver Prostate Centre, Department of Urologic Sciences, University of British Columbia, Vancouver, BC, Canada; Department of Medical Oncology, BC Cancer, Vancouver, BC, Canada; Vancouver Prostate Centre, Department of Urologic Sciences, University of British Columbia, Vancouver, BC, Canada; Michael Smith Genome Sciences Centre, BC Cancer, Vancouver, BC, Canada; Division of Urology, Department of Surgery, Huntsman Cancer Institute, University of Utah, Salt Lake City, UT, United States; Department of Internal Medicine, Spencer Fox Eccles School of Medicine, Salt Lake City, UT, United States; Department of Oncological Sciences, Huntsman Cancer Institute, University of Utah, Salt Lake City, UT, United States; Department of Biomedical Informatics, Huntsman Cancer Institute, University of Utah, Salt Lake City, UT, United States; Department of Tumor Biology, H. Lee Moffitt Cancer Center, Tampa, FL, United States; Division of Oncology, Department of Medicine, Huntsman Cancer Institute, University of Utah, Salt Lake City, UT, United States

## Abstract

**Background:**

To determine the performance of a multi-gene copy number variation (MG-CNV) risk score in metastatic tissue and plasma biospecimens from treatment-naïve metastatic castration-resistant prostate cancer (mCRPC) patients for prediction of clinical outcomes.

**Methods:**

The mCRPC tissue and plasma cell-free DNA (cfDNA) biospecimen sequencing results obtained from publicly accessed cohorts in dbGaP, cBioPortal, and an institutional mCRPC cohort were used to develop a MG-CNV risk score derived from gains in *AR, MYC, COL22A1, PIK3CA, PIK3CB, NOTCH1* and losses in *TMPRSS2, NCOR1, ZBTB16, TP53, NKX3-1* in independent cohorts for determining overall survival (OS), progression-free survival (PFS) to first-line androgen receptor pathway inhibitors (ARPIs). The range of the risk scores for each cohort was dichotomized into “high-risk” and “low-risk” groups and association with OS/PFS determined. Univariate and multivariable Cox proportional hazards regressions were applied for survival analyses (*P* < .05 for statistical significance).

**Results:**

Of 1137 metastatic tissue-plasma biospecimens across all cohorts, 699/1137 were treatment-naive mCRPC (235/699 metastatic tissue; 464/699 plasma-cfDNA), and 311/1137 were matched tissue-cfDNA pairs. In multivariable analysis, the MG-CNV risk score derived from metastatic tissue or in cfDNA was statistically significantly associated with OS with high score associated with short survival (hazard ratio = 2.65, confidence interval = 1.99 to 3.51; *P* = 1.35^−11^) and shorter PFS to ARPIs (median PFS of 7.8 months) compared with 14 months in patients with low-risk score.

**Conclusions:**

A molecular risk score in treatment-naïve mCRPC state obtained either in metastatic tissue or cfDNA predicts clinical survival outcomes and offers a tumor biology-based tool to design biomarker-based enrichment clinical trials.

## Introduction

Prostate cancer will account for more than 34 000 deaths in US males^1^ and more than 325 000 deaths worldwide^2^ in 2024. Therapeutic advances in metastatic hormone-sensitive prostate cancer (mHSPC) have evolved rapidly,^3^ but drug resistance and progression to metastatic castrate-resistant prostate cancer (mCRPC)^4^ occurs inevitably. Current mCRPC treatments include chemotherapeutic agents (docetaxel, cabazitaxel, and mitoxantrone); androgen receptor pathway inhibitors (ARPIs); PARP-1/2 inhibitors;^5^^,^^6^ radium-223,^4^^,^^7-10^ and lutetium-177 (^177^Lu)-PSMA-617.^11^ Choosing therapies for individual patients is empiric, as validated molecular predictors of survival or therapy response in mCRPC are lacking.^12^ In mCRPC prognostic and predictive biomarkers previously investigated include PSA,^12^ hemoglobin, lactate dehydrogenase and alkaline phosphatase levels,^12^ βIII-tubulin,^13^ CTCs,^14^^,^^15^  *AR-V7*, *AR-V9* splice variants,^16-21^ plasma tumor fraction (circulating tumor DNA-ctDNA),^22-31^ and plasma cell-free DNA (cfDNA),^32-34^ among others. Metastatic tissue-based profiling in mCRPC for biomarker development is informative for biomarker development but challenging to implement in clinical practice due to insufficient tumor yield^35^ and the invasive nature of biopsies. The use of ctDNA in plasma cfDNA fractions is actively studied in the mCRPC state^25^^,^^29^^,^^31^^,^^36-44^ to assess how well plasma-captured somatic alterations represent tissue-based genomic “truth sets” for biomarker comparisons. Previous attempts to determine concordance of plasma cfDNA-based somatic alterations with metastatic tissue have yielded varied results depending on the type of genomic alteration compared and the sequencing platform used. Low ctDNA in plasma or low tumor purity in tissue can drive discordance; true concordance requires sufficient tumor material in both analytes. Table S1 details recent tissue-plasma concordance studies in advanced prostate cancer.

In mCRPC state, copy number variations (CNVs) are common molecular alterations,^45-47^ and detection of cfDNA-based CNVs in plasma can be performed using low-pass coverage. CNV concordance between plasma and metastatic tissue has been reported to be high^38^^,^^48^^,^^49^ in mCRPC biospecimens obtained during or after first-line mCRPC therapy (Table S1). Because clonal evolution occurs with disease progression^50^ and changes in molecular landscapes due to treatment-induced lineage plasticity (TILP) can influence molecular alterations, we attempted to determine somatic CNVs in treatment-naïve mCRPC plasma and metastatic tissue biospecimens as potential classifiers of clinical outcomes. A second objective was to compare CNVs in plasma-metastatic tissue pairs in treatment-naïve mCRPC state. To accomplish these objectives, the current study used multiple treatment-naïve mCRPC datasets that were publicly accessed. The performance of a previously reported cfDNA derived multi-gene CNV risk score (MG-CNV)^51^ associated with mCRPC outcomes as a molecular classifier of outcomes was determined in these independent datasets. The 11 genes include somatic CNV gains in *AR, MYC, COL22A1, PIK3CA, PIK3CB,* and *NOTCH1* and loss in *TMPRSS2, NCOR1, ZBTB16, TP53,* and *NKX3-1.*

## Methods

### Description of treatment-naïve mCRPC databases

This study probed 3 published datasets that were publicly available for which no patient contact was made. The first dataset was a treatment-naïve mCRPC cohort database with concurrently collected matched serial plasma^51^ and metastatic site biopsy pairs^45^^,^^46^ before and after 12 weeks of abiraterone acetate/prednisone (AA/P) treatment. These deidentified metastatic tissue datasets henceforth referred to as “PROMOTE” (Prostate Cancer Medically Optimized Genome-Enhanced Therapy; https://clinicaltrials.gov/ identifier NCT #01953640) database are deposited in public databases (dbGaP), and the clinical outcomes for all PROMOTE biospecimen were obtained from published reports^51^ along with individual patient plasma CNV status of the 11 genes for both visits. The second dataset was a mCRPC tissue dataset in cBioPortal (https://www.cbioportal.org/), henceforth referred to as “SU2C/PCF” (Stand Up to Cancer-Prostate Cancer Foundation Dream Team).^47^ The third dataset was mCRPC cfDNA sample and clinical dataset obtained from the Vancouver Prostate Cancer and BC Cancer,^31^ henceforth referred to as “VPC” dataset. Details of each dataset are provided in Supplementary Methods.

### Statistical analysis of copy number variation

The primary processing of tumor and germline exome DNA analysis used a series of docker/singularity snakemake workflows. Details of all workflows and GitHub links are provided in Supplementary Methods. In brief, CNV calls in the PROMOTE metastatic tissue data were determined by GATK best practice workflow, whereas the PROMOTE plasma CNV calls were directly extracted from published results.^51^ Linking of CNV status for tissue/plasma pairs with clinical outcomes is provided in Table S2 with details under Supplementary Methods. Similarly, CNV calls in the SU2C/PCF dataset were directly extracted from cBioPortal. In the VPC dataset,^31^ CNV calls were available for 8/11 genes of interest. Calculation of the MG-CNV risk score and definitions of “high” risk and “low” risk MG-CNV scores are detailed in Supplementary Methods.

## Results

**Figure 1.**
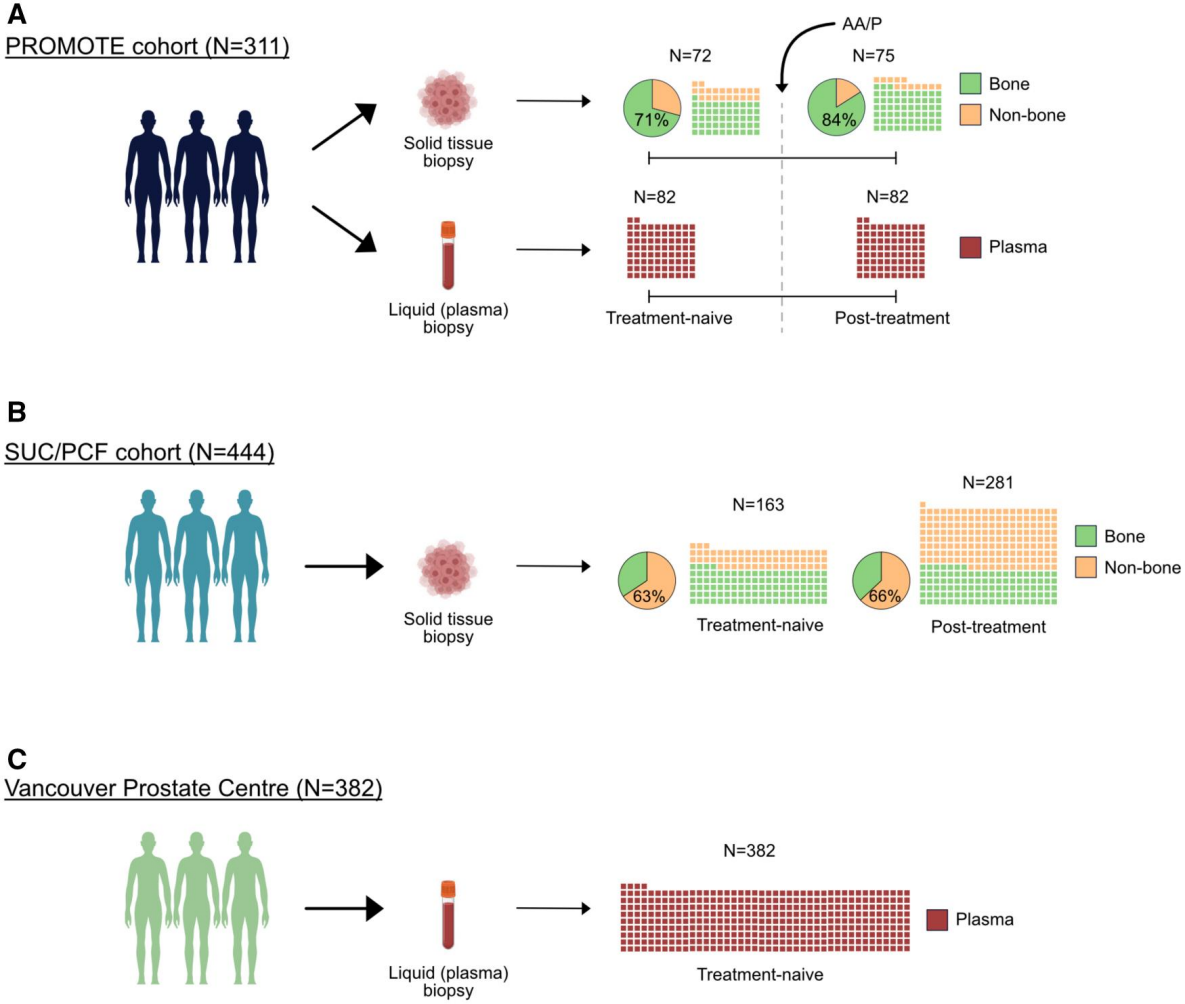
Study cohorts overview. **A**) Details of the PROMOTE cohort. Number of metastatic castrate-resistant (mCRPC) patients with concurrent solid tissue and liquid (plasma) biopsy collected at “treatment-naive” and at 12 weeks “post-treatment” with AA/P along with the distribution of metastatic tissue sites biopsied at both time points. **B**) Details of the SU2C/PCF cohort. Number of solid tissue biopsies and the distribution of metastatic tissue sites. Samples not exposed to AR signaling inhibitor (abiraterone acetate or enzalutamide) are shown as “treatment-naive” and those exposed are shown as “post-treatment.” **C**) Details of the Vancouver Prostate Cancer cohort. All liquid (plasma) biopsy are collected before to any exposure to AR signaling inhibitor. Abbreviations: PROMOTE = PROstate cancer Medically Optimized genome enhanced ThErapy; AA/P = abiraterone acetate (AA)/prednisone (P); SU2C/PCF = Stand Up to Cancer-Prostate Cancer Foundation Dream Team.

The distribution of a total of 1137 mCRPC biospecimen across 3 independent cohorts is shown in Figure 1. The dbGaP-based PROMOTE dataset contained 311 biospecimens, which includes 2 serial collections of matched metastatic tissue and plasma-based pairs (Figure 1, A). cBioPortal (SU2C/PCF Dream Team) cohort contained 444 mCRPC metastatic tissue biospecimen (Figure 1, B), and a third mCRPC cohort from the Vancouver Prostate Centre (VPC) and BC Cancer registry contained 382 plasma biospecimens (Figure 1, C). Figure S1 represents the treatment-naïve biospecimen analyzed.

For metastatic tissue, we first established thresholds for CNV calls in the PROMOTE dbGaP metastatic tissue sequencing results. Thresholds to call copy number gain or loss from WES data varies from a lenient log2 ratio of 0.1^49^ to a more stringent log2 ratio of 0.4.^52^ We compared CNV frequency in the 11 genes of interest between PROMTOE tissue dataset and 6 independent public mCRPC datasets available in cBioPortal (Figure S2). Based on these comparisons, we established the most optimal log2 ratio cutoff for gains and loss calls in the PROMOTE dataset to be log2 ratio ±0.5 (Supplementary Materials). PROMOTE CNV calls are provided in Table S3.

**Figure 2.**
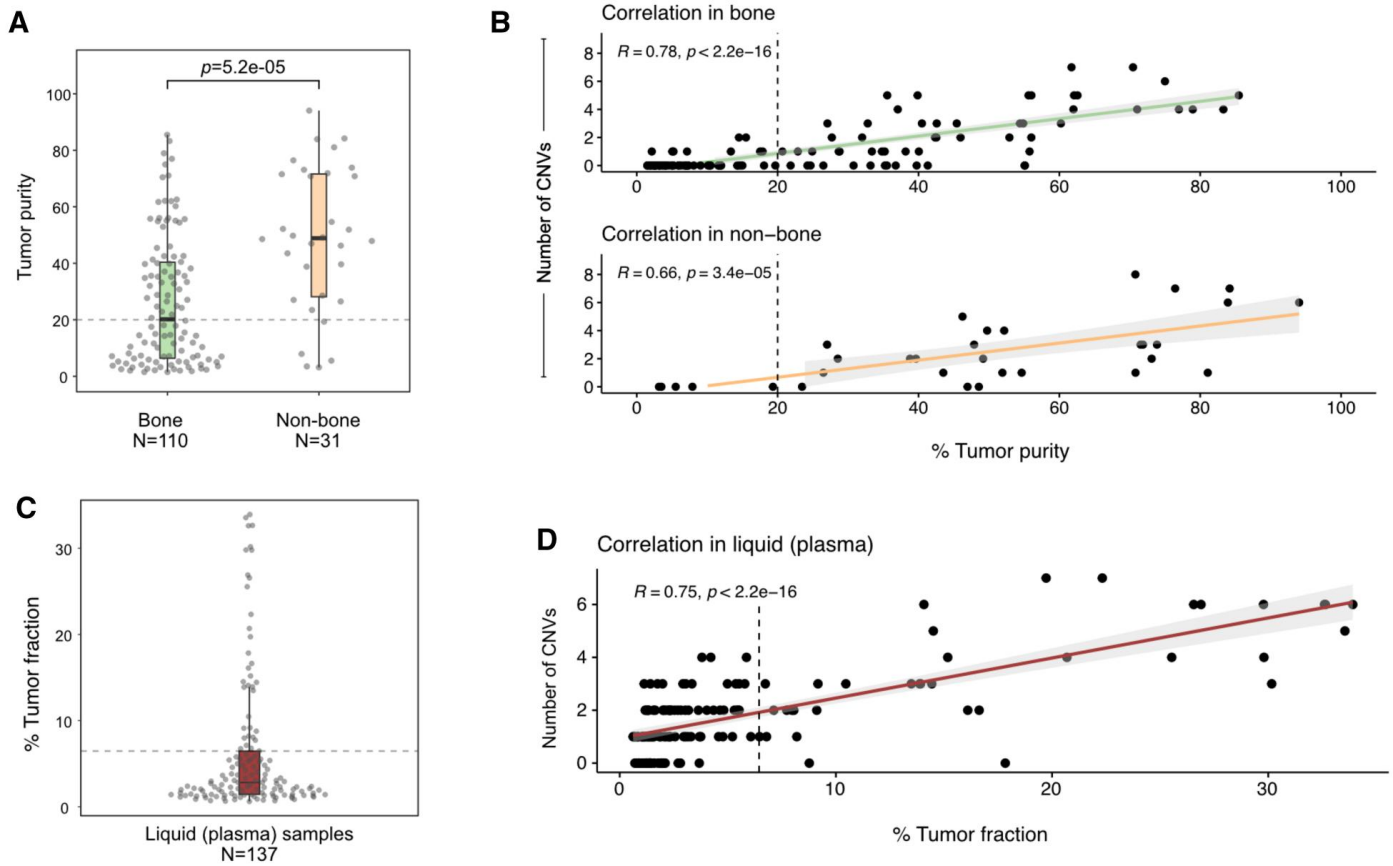
Association between CNV calls with DNA purity and tumor fraction. **A**) Comparison of tumor DNA purity in all the solid tissue biopsies collected from bone (*N* = 110) and non-bone sites (*N* = 31) that was detected to be statistically greater in non-bone sites (2-tailed student *t* test). Each point represents a single sample collected either at treatment-naive or post-treatment. Samples with DNA purity above 20% (indicated by horizontal dashed line) were considered high tumor purity, whereas samples with 20% or lower were considered low tumor purity. **B**) Correlation of the number of CNVs detected in the 11 genes of interest with tumor DNA purity in bone (top) and non-bone (bottom) tissue sites. The number of samples are equal to those in panel A. **C**) Distribution of tumor fraction (circulating tumor DNA (ctDNA) percentage) in all liquid (plasma) biopsies collected samples. Each point represents a single sample collected either at treatment-naive or post-treatment. Samples with tumor fraction above the third quartile (indicated by horizontal dashed line, ctDNA = 6.47%) were considered samples with high tumor fraction, and samples with below or equal the value were considered low tumor fraction. **D**) Correlation of the number of CNVs detected in the 11 genes of interest with tumor fraction. The number of samples are equal to those in panel C.

We next evaluated the impact of tissue DNA tumor purity on CNV frequency in metastatic tissue and matched plasma specimens. Figure 2 highlights the association between CNV calls and metastatic tissue DNA purity and with plasma tumor fraction (ctDNA). We set the tissue tumor DNA purity threshold at 20%, with biospecimen less than 20% tumor DNA purity labeled as “low” and 20% or greater as “high,” per published reports.^47^ Distribution of tumor DNA purity in solid tissue biopsies collected from bone (*N* = 110) and non-bone sites (*N* = 31) is shown in Figure 2, A. Figure 2, B shows a positive correlation of the number of CNVs detected in the 11 genes with tumor DNA purity in bone (R = 0.78; *P* < 2.2^−16^) and non-bone (R = 0.66; *P* = 3.4^−5^) tissue sites. Figure 2, C shows the distribution of tumor fraction (ctDNA) percentage in PROMOTE plasma samples, and Figure 2, D shows correlation of the number of CNVs detected in the 11 genes of interest with tumor fraction (ctDNA) (R = 0.75; *P* < 2.2^−16^). These results are detailed in Supplementary Materials.

**Figure 3.**
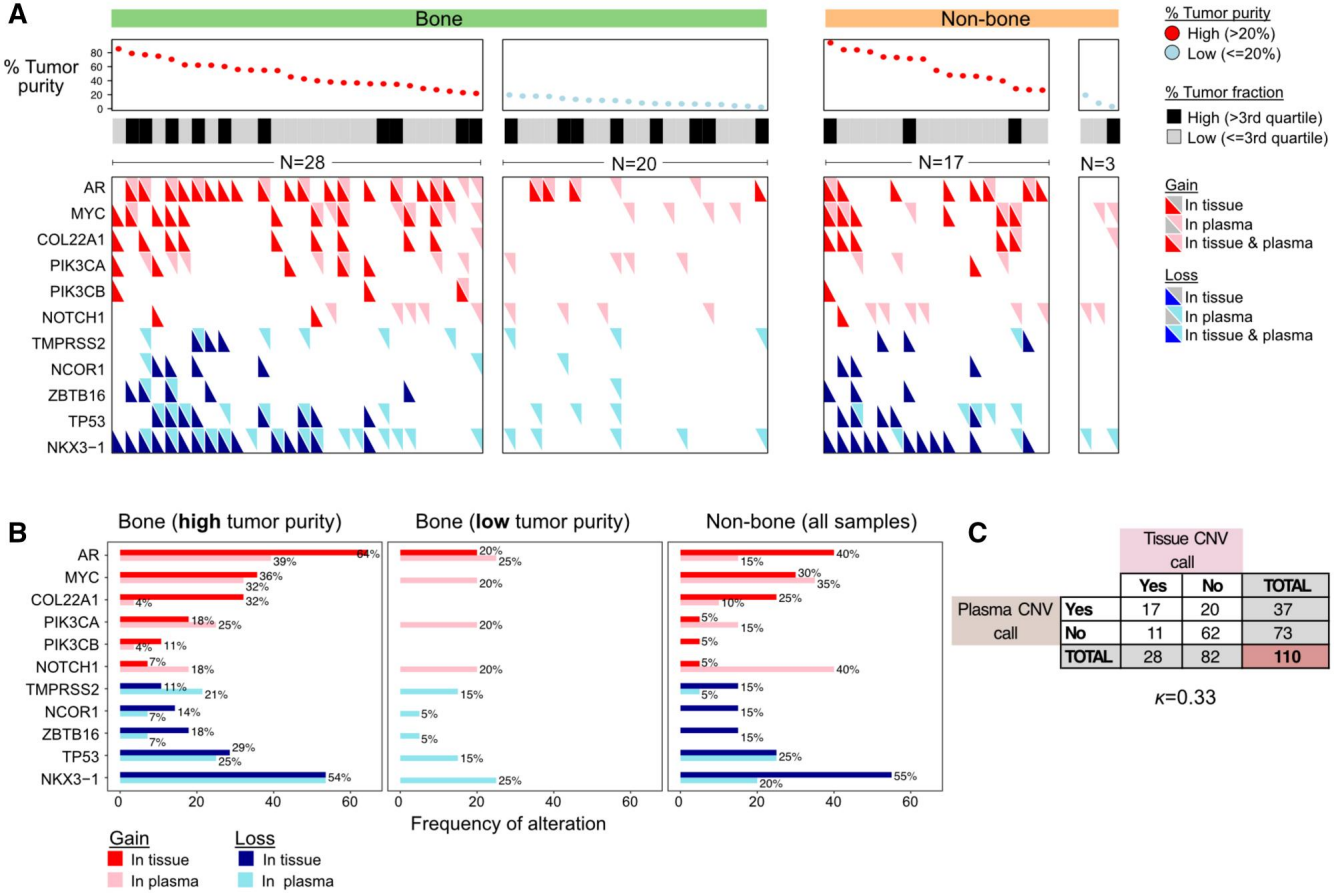
Landscape of copy number variations (CNVs) detected in the treatment-naive matched tissue-plasma PROMOTE biospecimen. **A**) Heatmap showing individual patients in columns and the CNV calls for the 11 genes in rows in the matched tissue-plasma treatment-naive biopsies. CNV calls in the tumor tissue are represented by red triangles for CNV gains and blue triangles for CNV loss. Matching CNV calls in liquid (plasma) biopsy is represented by light pink triangles for gains and light blue triangles for CNV loss. Patients are grouped based on tissue biopsies performed in bone and non-bone sites and based on DNA tumor purity in metastatic tissue (≤ or >20%). The categorization of matched liquid (plasma) ctDNA fraction to high- or low-tumor fraction is based on the third quartile range (ctDNA = 6.47%). **B**) Frequency of CNVs detected in paired tissue (red/blue bars) and plasma (faint pink/blue bars) for the 11 genes of interest with red bars for CNV gains and blue for CNV loss. Frequency is grouped based on metastatic sites and DNA tumor purity. **C**) Contingency table of CNVs detected in tissue and liquid (plasma) biospecimen limited to bone-based biopsy samples with high tumor purity and liquid (plasma) biopsy samples with high tumor fraction (*N* = 10). *K—*Kappa statistic (degree of agreement between tissue and plasma) is indicated below the contingency table.

The landscape of CNV tissue-based calls for all 11 CNVs in the 68 treatment-naive samples matched with the CNVs from concurrently matched cfDNA pairs is shown in Figure 3, A. Across all the biospecimens, *AR* gain and *NKX3-1* loss were the most altered CNVs in tissue and plasma cfDNA (Figure 3, B). The degree of agreement for CNV calls between matched treatment-naive tissue and cfDNA pairs with high tumor purity and high tumor fraction as measured using a kappa statistic was 0.33, indicating a fair degree of agreement (Figure 3, C). Further details of the treatment-naive landscape of tissue-plasma pairs based on site and tumor purity are provided in Supplementary Materials. The 12-week post-treatment landscape of CNV calls in 74 matched metastatic tissue/cfDNA pairs for all 11 CNVs is shown in Figure S3.

**Figure 4.**
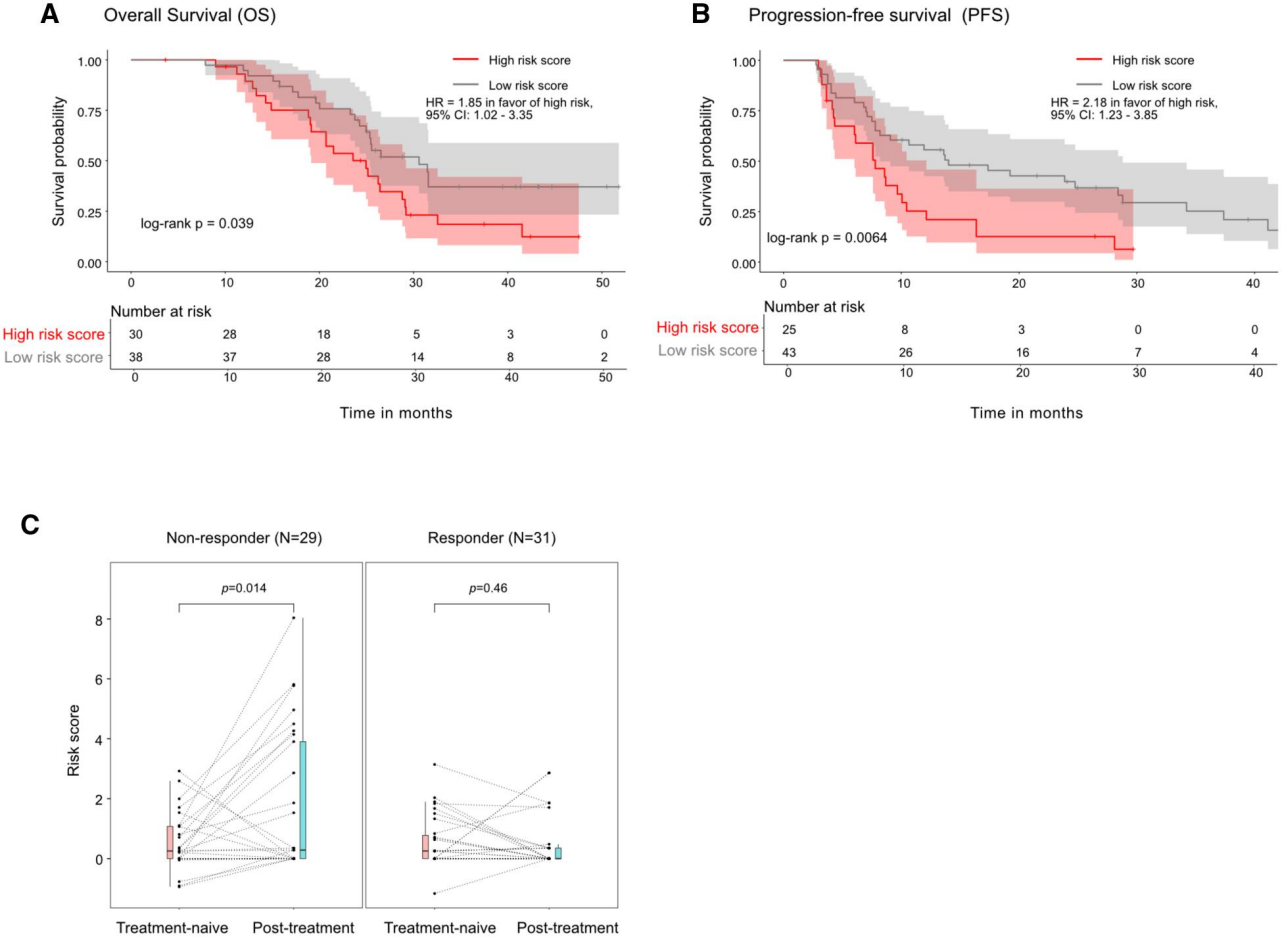
Association of the PROMOTE tissue-based multi-gene risk score with overall survival (OS) and progression-free survival (PFS).

*MG-CNV risk score and clinical outcomes in the matched tissue biospecimen:* Our study goal was to evaluate the clinical performance of a previously reported cfDNA-based MG-CNV risk score^51^ in metastatic tissue. Table S4 lists the metastatic tissue-based risk scores from treatment-naive and post-12-week treatment time-points, as calculated for the clinical outcomes of overall survival (OS) and progression-free survival (PFS) for AA/P therapy. Clinical response to AA/P therapy of each research subject was performed prospectively at 12 weeks and is also listed. The range of the calculated tissue-based risk scores for predicting OS in the treatment-naive visit was between -0.11 and 4.01 with a median of 0.37, and 30/68 patients had high-risk score values (above the median) with a median survival of 24.9 months (range = 3.7-47.5 months) compared with patients with low-risk scores (*n* = 38/68) with a median survival of 30.6 months (range = 7.9-51.8) months (*P* = .039). The range of the calculated risk score for predicting PFS to AA/P was between -1.16 and 3.14 (median = 0.25). Patients with high-risk scores showed a median PFS of 7.8 months compared with 14 months in patients with low-risk scores. Kaplan-Meier survival plots for OS and PFS, respectively, in treatment-naïve mCRPC patients based on the tissue MG-CNV risk score (high vs low) are shown in Figure 4, A and B. To determine if the metastatic site and DNA tumor purity were predictive of OS/PFS, we explored the impact of metastatic site biopsy (bone vs non-bone) and high vs low DNA tumor purity to predict OS/PFS and found no impact of these factors on clinical outcomes (Figure S4). Figure 4, C demonstrates changes between treatment-naive and 12-week post-treatment risk score for each individual patient based on response at 12 weeks. Patients with primary resistance to AA/P (defined as nonresponders at 12 weeks) were observed to have a statistically significant increase in the risk score. Details of pharmacodynamic changes in the MG-CNV tissue-based risk score after 12 weeks of AA/P therapy are provided in Supplementary Materials.

**Figure 5.**
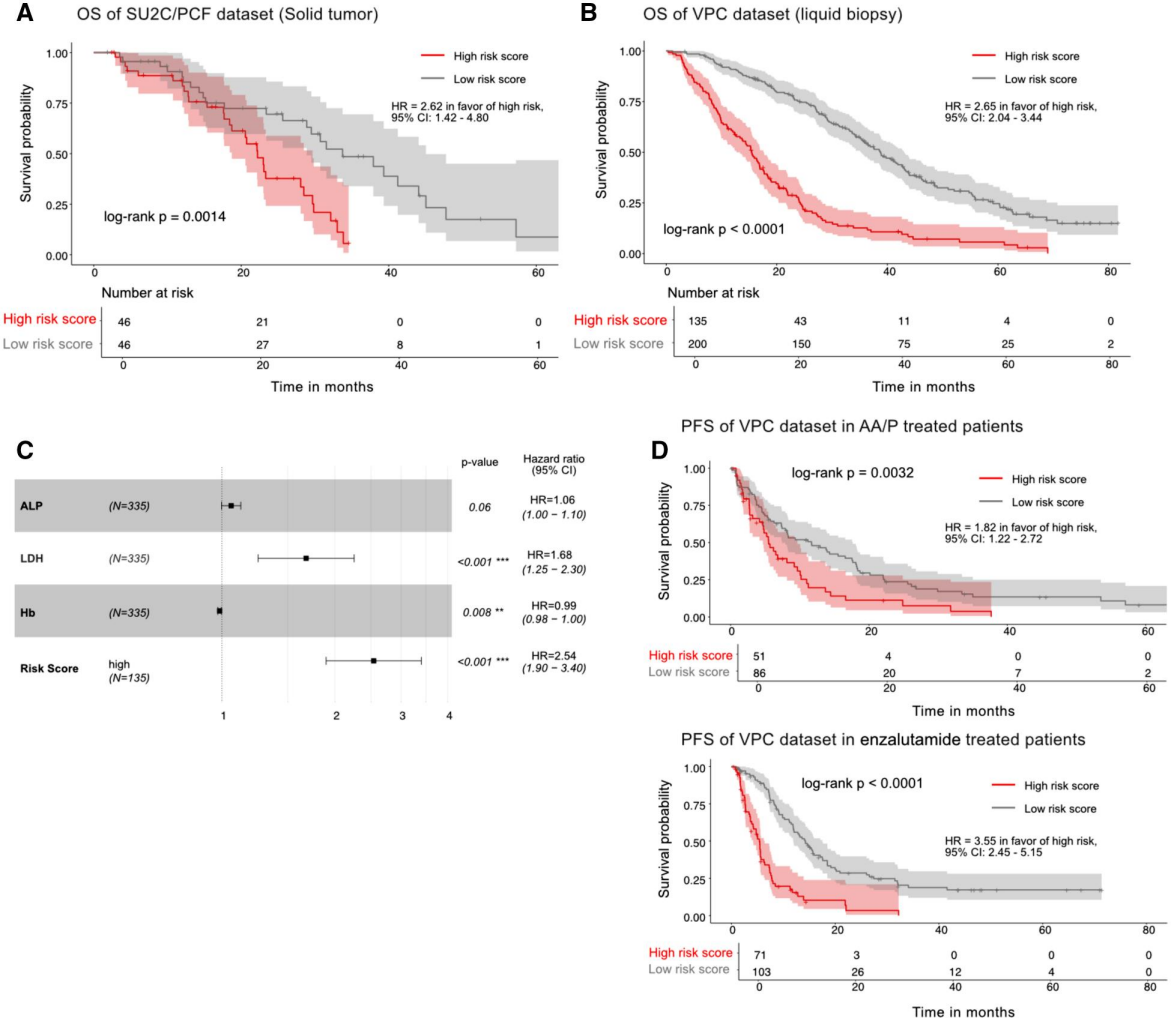
Association of the multi-gene risk score with overall survival (OS) and progression-free survival (PFS) in 2 independent cohorts of tissue and plasma biospecimen. **A**) Multi-CNV risk score association with OS calculated from 11 CNVs in treatment-naive solid tissue mCRPC patients (*N* = 98) from cBioportal (StandUpToCancer-Prostate Cancer Foundation study). **B**) Multi-CNV risk score association with OS calculated using 8-gene CNV panel in treatment-naive mCRPC patients (*N* = 335) from the Vancouver Prostate Centre (VPC) and British Columbia Cancer dataset. **C**) Multivariable Cox regression hazard ratio analysis includes 3 clinical (laboratory) factors and the multi-CNV risk score in the University of British Columbia liquid (plasma) biopsy dataset; (ALP = serum alkaline phosphatase; HgB = hemoglobin; LDH = serum lactate dehydrogenase). **D**) Multi-CNV risk scores association with PFS from University of British Columbia-Prostate Cancer Institute dataset calculated using the 8-gene CNV panel in treatment-naive mCRPC patients (*N* = 137) subsequently treated with abiraterone acetate/prednisone (AA/P) (top) and enzalutamide (*N* = 174) (bottom).

We applied the same CNV call criteria to the SU2C/cBioPortal cohort to calculate the MG-CNV risk score. The risk score ranged from -0.45 to 3.59, with a median of 1.94 (Table S5) with 46/92 patients classified in the “high-risk” category (above the median) and 46/92 as low-risk patients. We observed that patients with high-risk score had a lower (median) survival of 22.2 months compared with patients with low-risk score at 33.7 months (Figure 5, A). Detailed results for the SU2C/cBioPortal cohort are provided in the Supplementary Materials, which also include results of two integrative gene transcription scores for predicting clinical outcomes, an *AR score* and a *NEPC score.*  Table S5 lists the individual AR and NEPC scores for the treatment-naïve biospecimens. No association with overall survival for either score was observed (Figure S5).

Finally, we determined the performance of the MG-CNV score in the VPC cfDNA specimen set. Table S6 provides results for each individual patient’s risk score calculated for OS and PSA-PFS in this cohort. For the overall survival endpoint, of the 335 patients 135 patients were classified as “high-risk” with a median survival of 15.5 months compared with the 200 patients classified as “low-risk” with a median survival of 38.8 months (Figure 5, B). In Figure 5, C we show that the highest hazard ratio (HR) for survival was observed for the MG-CNV risk score (HR = 2.64; range = 1.88-3.51; *P* = 1.35 × 10^−11^) at the multivariable level after adjusting for known clinical prognostic factors (Table 7), and Figure 5, D illustrates the survival curves for AA/P and enzalutamide treatments based on risk scores and PSA-PFS endpoint. Further detailed results for this cohort are provided under Supplementary Materials.

## Discussion

The primary goal of this multi-independent cohort study was to evaluate the performance of a MG-CNV risk score in treatment-naïve mCRPC patients as a molecular classifier of survival and therapy response for first-line ARPIs. The ability of a molecular classifier in plasma to match its clinical performance in metastatic tissue increases the acceptability for its application; therefore, we also determined the MG-CNV risk score performance in matched tissue-plasma pairs obtained before and after first-line AA/P mCRPC therapy. We observed that the MG-CNV risk score was able to prognosticate survival in concurrently obtained metastatic tissue-cfDNA biospecimen pairs (Figure 4, A), in addition to an independent metastatic tissue cohort (Figure 5, A) and an independent cfDNA cohort (VPC) (Figure 5, B), where it outperformed non-tumor-specific prognostic clinical factors in multivariable models (Figure 5, C). The MG-CNV risk score provides a molecular classifier for prognostication, which is currently lacking in this stage, and potentially identifies high-risk score patients for developing intensified or novel therapeutic interventions. We also observed that a high MG-CNV risk score is associated with a shorter duration of therapy response to AA/P, using either tissue (Figure 4, B) detected CNVs or cfDNA detected CNVs (Figure 5, D). Although these observations confirm initial results of using this as a classifier for AA/P treatment response,^51^ the MG-CNV risk score was also associated with shorter PSA progression-free survival response to enzalutamide therapy (Figure 5, D). Validated molecular markers that inform ARPI therapy response in mCRPC clinical practice are currently lacking, and this score offers a potential classifier for designing novel enrichment-type biomarker-based study designs in future. Finally, the risk score attempts to develop pharmacodynamic molecular events to therapy, in line with the Prostate Cancer Working Group-3 (PCWG-3) recommendations, which emphasize “serial biologic profiling using tumor samples from biopsies, blood-based diagnostics, and/or imaging is recommended to gain insight into mechanisms of resistance and to identify predictive biomarkers of sensitivity for use in prospective trials.”^53^ In this context, we observed an increase in the MG-CNV risk score in nonresponders at 12 weeks (Figure 4, C) using metastatic tissue as was previously reported from matched cfDNA biospecimen pairs.^51^ From a biological point of view, the MG-CNV-based risk score may at least partially reflect tumor burden.

We addressed several challenges while assessing the score performance in multiple cohorts including metastatic tissue-plasma biopsy-based CNV calls, tissue site biopsied, and tissue-DNA purity obtained because they may confound CNV frequencies. Based on these tissue tumor purity metrics, we considered different thresholds to establish rigorous thresholds for CNV calls. Our attempt was to mitigate the impact of false positive CNV calls in tissue by using lenient log2 ratio thresholds and to obtain a consensus on CNV call frequencies for the 11 genes as closely as possible across previously published 6 mCRPC datasets in cBioPortal (Figure S2), which led us to establish a log2 ratio threshold of >/< 0.5 for gain/loss. For plasma biopsies, the tumor fraction (ctDNA) in plasma biospecimen can be low and increase the false negative CNV calls. We observed that ctDNA in the PROMOTE dataset (Figure 2, C) correlated well with CNV calls (Figure 2, D) and in this cohort set a ctDNA threshold of 6.47% and above to classify “high” vs “low” tumor fraction (Figure 2, D). We observed that for high-DNA tissue purity and plasma ctDNA tissue-plasma pairs the kappa statistic, a measure used for assessing interobservational reliability of agreement, was 0.33 (Figure 2, C), indicating a fair agreement. These observations have clinical relevance, as increasingly CLIA-CAP commercial laboratory NGS tests in metastatic biopsies do not adequately report tumor tissue DNA purity for CNVs/SNV calls, which can introduce bias in the results for clinical applications. Taken together in comparative concordance studies, both ctDNA fraction and tissue tumor DNA purities are critical to know to determine the positive and negative predictive values (PPV/NPV) for plasma cfDNA based detection of CNV loss during clinical application.

Despite the methodological approaches and multiple independent cohorts used, our study has a few limitations. First, we note that there are differences in sequencing approaches applied to metastatic tissue and cfDNA, which can affect CNV calls. Metastatic tissues in the different cohorts were sequenced using WES in PROMOTE and SU2C/PCF, whereas plasma samples were sequenced via a deep targeted capture-based approach in the VPC cohort, and PROMOTE plasma cfDNA was sequenced using low-pass WGS (0.5× coverage). Small focal CNVs (eg, *BRCA2* homozygous deletion) are often missed by low-pass WGS/WES approaches, whereas large chromosomal-arm changes are typically readily detected. In addition, the WES has high coverage at exon regions, but it misses the main component of introns in target genes. Therefore, in addition to coverage, the size and number of exons may affect CNV calls for solid tissues. We note that in our approach in the PROMOTE tissues we used GATK package, whereas PROMOTE cfDNA biospecimen used a gene-centric normalization method^51^ for CNV calls in which the sequencing covers entire gene regions including both exons and introns. Although the coverage is low, the unique gene read count across target genes is still sufficient for comfortable CNV calls. These differences in CNV calling algorithms and differences in coverage and sequencing approach, log2 ratio cutoffs for CNV calls can affect postsequencing results. Despite these differences, the MG-CNV score demonstrated prognostic value in all mCRPC cohorts. Although our analysis demonstrated the prognostic value of the MG-CNV risk score across multiple cohorts, we acknowledge the absence of key clinical variables such as age, disease burden, and performance status in these public datasets. These factors may affect survival outcomes, and their unavailability prevented us from adjusting for these confounders. Finally, these results capture the heterogeneity at one specific point of cancer progression. It is not clear if results apply to all points of the cancer continuum in the spectrum of mCRPC progression and after different drug treatments preceding mCRPC state because the therapeutic landscape for mHSPC has expanded dramatically and clonal evolution complexities^50^ may result in TILP. Evaluating clonal diversification before developing state-specific molecular signatures is critical, but currently unknown.

Barring detection of tissue or plasma detected homologous recombinant pathway mutations^54-56^ that apply to a small proportion of mCRPC patients, biomarker-based therapeutic targeting is lacking. In this context the performance of a plasma cfDNA-based MG-CNV score has potential for prospective testing as a prognostic and potential predictive biomarker in the biomarker-enrichment type of clinical trial designs, which is a critical step before translation into clinical applications. We have recently reported how a plasma MG-CNV risk score classifier may be prospectively validated in mCRPC by exploring in a simulation study a plasma-based MG-CNV score to design a biomarker enrichment therapeutic strategy.^57^ The simulation study provides a template for next steps on our reported 11-gene MG-CNV score to be used as a classifier that refines mCRPC state therapeutics based on tumor-biology in future.

## Supplementary Material

pkaf025_Supplementary_Data

## Data Availability

Raw data analyzed in the course of the study are available on dbGaP (phs001141; PRJNA325181) or cBioPortal (https://cbioportal-datahub.s3.amazonaws.com/prad_su2c_2019.tar.gz). Analysis scripts are available at https://github.com/HuntsmanCancerInstitute/Workflows/ and https://github.com/zakiF/PublishedPapers/ProstatePROMOTE. Processed CNV calls are available as Tables S3-S6.
