# Supplementary material for: Multi-gene risk score for prediction of clinical outcomes in treatment-naïve metastatic castrate-resistant prostate cancer
Source: JNCI Cancer Spectr. 2025 Feb 28;9(2):pkaf025. doi: 10.1093/jncics/pkaf025 (PMC11954629; doi:10.1093/jncics/pkaf025)
Supplement: pkaf025_Supplementary_Data [file pkaf025_supplementary_data.zip › 04_Supplementary_Materials.docx]

# **Supplementary Materials**

Table of Contents

[Supplementary Methods 2](#_Toc187399250)

[Supplementary Results 13](#_Toc187399251)

[Supplementary Tables Legend 25](#_Toc187399252)

[Supplementary Figures 29](#_Toc187399253)

[Supplementary Figure 1: Patient biospecimen filtering in all cohorts 30](#_Toc187399254)

[Supplementary Figure 2: Frequency of Copy Number Variations (CNVs) in metastatic tissue datasets for 11 genes of interest 32](#_Toc187399255)

[Supplementary Figure 3: Landscape of Copy Number Variations (CNV)s in the 12-week post treatment biospecimen dataset from PROMOTE 34](#_Toc187399256)

[Supplementary Figure 4: Association of metastatic tissue tumor site and DNA tumor purity with overall survival (OS) and progression free survival (PFS) in treatment-naive PROMOTE tissue biospecimen 36](#_Toc187399257)

[Supplementary Figure 5: Association of AR score and NEPC score with overall survival (OS) in treatment naive cBioPortal tissue biospecimen 38](#_Toc187399258)

# **Supplementary Methods**

**Supplementary Methods**

Three independent treatment-naïve mCRPC biospecimen cohorts with publicly available CNV sequencing data and clinical outcomes in metastatic tissue and plasma biospecimens were accessed. We determined the concordance of the MG-CNV-based risk score in matched, concurrently obtained metastatic tissue and plasma pairs in these cohorts when available before and after initiating first-line Androgen Receptor Pathway Inhibitors (ARPI) therapies.

**Description the publicly accessed dbGaP-accessed *“PROMOTE” study biospecimen cohort***

The “PROMOTE” (Prostate Cancer Medically-Optimized Genome-Enhanced Therapy) prospective cohort study was initiated in May 2013 after obtaining approval from Mayo Clinic Institutional Review Board (IRB). Accrual was completed in October 2015 and clinical follow-up continued until October 2018. All research subject enrolled in the trial provided a written informed consent approved by the IRB and at the time of enrollment had to have sub-castrate testosterone levels (less than 50 ng/dl) and the median PSA at the time of enrollment for this cohort was measured at 14.6 ng/ml (Range: 6.4-41.5 ng/ml). Complete details of the cohort have been previously reported [4]. Study procedures included a concurrently obtained treatment-naïve metastatic site biopsy and a blood draw (plasma biopsy) for characterizing molecular landscapes of the tumor prior to initiating abiraterone acetate and prednisone therapy (AA/P) therapy. Tumor tissue biopsies were collected from bone or soft tissue before initiation of AA/P. A second serial biopsy was performed 12-weeks after initiating AA/P, which also included a metastatic site biopsy and a plasma biopsy. Progression status at 12-weeks after initiating AA/P was determined per the recommendations of the Prostate Cancer Working Group-2 criteria (PCWG2) [5]. This included serum PSA, bone and CT imaging and symptom assessments using the Functional Assessment of Cancer Therapy-Prostate (FACT-P) scale performed at the same time (week 12) [5]. Treatment response was defined as the absence of PSA progression (as defined by the PSA Working Group Criteria); absence of any new bone lesion on bone scan and no radiological (RECIST 1.0) progression of nodal or soft tissue metastases. Bone scans at 12 weeks in which new lesions were detected were repeated with a follow-up bone scan at least six more weeks and if additional new bone lesions were observed at the second follow-up scan patient was deemed to have progression. Research subjects who met one of these criteria for progressive disease were defined as “non-responders” after 12 weeks of drug exposure. Results of molecular landscapes based on metastatic tissue and plasma biopsies for both visits have been previously reported extensively [1, 2, 4]. Sequencing results for the “PROstate cancer Medically Optimized genome enhanced ThErapy (PROMOTE)” project (phs001141; PRJNA325181) were accessed from dbGaP after institutional IRB approval.

This linking of the de-identified publicly accessed genomic and clinical data for the “PROMOTE” cohort [1, 4] with concurrently collected matched tissue-plasma [2] pairs at the two time points was performed using publicly available sources, without any patient contact. Sequencing details linking the de-identified tissue and cfDNA pair IDs alongside the clinical information are provided in “**Supplementary Table 2**”. **Figure 1A** illustrates the study schema and the number of matched plasma-metastatic biopsy pairs available in each of the two serial visits accessed for analysis (N=311), along with the number of metastatic tissue biopsies performed in bone versus non-bone sites in each serial visits. Of the 82 cfDNA biospecimen with sequencing data available [2] there were 72 matched metastatic tissue biospecimen pairs with sequencing data available for analysis at the treatment-naive-treatment time-point in dbGaP. At the 12-week post AA/P treatment time-point of the 82-post treatment cfDNA biospecimen, 75 matched metastatic tissue biospecimen sequencing data were available in dbGaP (**Figure 1A**). Of the 72 treatment-naive-treatment and 75 post-treatment metastatic tissue samples, 4/72 and 1/75 were excluded from further analysis due to poor sequencing coverage (details listed **under “DNA Alignment** and **Quality Filtering” section in Supplementary Methods** and in **Supplementary Table 2**). Thus, in the PROMOTE cohort, analysis is reported for 68 treatment-naive-treatment and 74 post-treatment biopsies matched metastatic-tissue and cfDNA biospecimen pairs (**Supplementary Figure 1A**). **Figure 1A** also details the metastatic biopsy sites for the 72-treatment-naive-treatment metastatic tissue biospecimen sequencing data include 48/72 from bone and 17/72 from non-bone sites. In the 75 post-treatment sequencing data, 62/75 were performed in bone tissue and 13/75 in non-bone metastatic tissue.

To link the de-identified clinical and plasma to metastatic tissue genomic data in dbGaP database at the individual patient-level, the online de-identified metastatic tissue-plasma biopsy patient identification codes from dbGaP and online sources for both visits are provided in **Supplementary Table 2.** The table list the results of the individual biospecimen IDs linked with the metastatic tissue biopsy from dbGaP [1] with the plasma biopsy IDs [2] and the individual patient outcomes for overall survival (OS), progression-free survival (PFS) and treatment responses. **Supplementary Table 2** also shows ctDNA and metastatic tissue tumor content for each biospecimen (metastatic tissue and plasma biopsy) for both visits. To obtain the CNV profiles of the 11 genes we processed the publicly available metastatic tissue WES data for both visits through our bioinformatics workflow as detailed below.

***Metastatic tissue analysis of the PROMOTE study cohort***

***Data Access from dbGAp***: De-identified patient exome datasets used in this study were downloaded from the dbGaP “PROstate cancer Medically Optimized genome enhanced ThErapy (PROMOTE)” project (phs001141; PRJNA325181). 229 Short Read Archive (SRA) files (82 germline, 72 treatment-naive-treatment (Visit 1) tumors, 75 post-treatment (Visit 2) tumors) were downloaded from the dbGaP server (**Supplementary Table 2**) after University of Utah IRB approval (#IRB_00135608).

***Primary Bioinformatics analysis***: The primary bioinformatic analysis in this study utilized a series of docker/singularity containerized snakemake workflows to process each patient’s tumor and germline/normal exome DNA and datasets. These optimized workflows are publicly available from Huntsman Cancer Institute’s GitHub repository [GIT] accessible at the GitHub repository: <https://github.com/HuntsmanCancerInstitute/Workflows/>. In brief, these workflows follow current best practices to: (i) align sequencing data to the human reference GRCh38 build, (ii) call and annotate germline single nucleotide variants (SNV) and insertion/deletion (INDEL) variants, (iii) identify somatic copy number variation (CNV) and (iv) calculate a variety of quality control (QC) metrics for unique observation alignment read depth, sample variant and gender concordance. Execution of the workflows is coordinated using the USeq 9.3.0 TNRunner application [USEQ]. The downloaded SRA files were transferred to a private Amazon Web Services bucket and subsequently downloaded to Linux servers in the protected domain at the University of Utah’s Center for High Performance Computing. Each SRA file was converted to Illumina 101bp paired-end fastq using the SRA Toolkit 3.0.1 [SRA].

***DNA Alignment and Quality Filtering:*** The DnaAlignQC workflow aligns exome fastq sequence reads to GRCh38 (bwa-mem2 2.2.1 [BWA]), filters the alignments for unique mapping (MQ>=13) and removed duplicates (Samtools 1.9 [ST]). Five of 229 samples with a unique read coverage of <12x at 95% of capture design defined regions of interest were designated as low quality and excluded from this analysis (**Supplementary Table 2**). The mean on target coverage across all dbGap downloaded samples were 187x.

***Germline Variant Calling:*** Germline variant calls are needed for somatic copy analysis and was accomplished by deploying two workflows utilizing tools from GATK [GATK G]. The first recalibrates base alignment scores and calls haplotypes to generate a g.vcf file for each normal exome sample. The second merges the g.vcf files from all samples, jointly calls the genotypes, recalibrates the snv and indel scores separately, and applies two quality filters. The first is a GATK VQSR tranche of 99.9. The second is a collection of hard cutoffs requiring a variant QUAL >= 20, read depth DP >= 10, allele fraction >= 0.05, a genotype quality >= 20, and number of unique alternative observations >=3 using the USeq JointGenotypeVCFParser.

***Somatic Copy Ratio Analysis (CRA):*** Somatic copy number variations were identified using a CopyAnalysis snakemake workflow based around GATK’s best practice protocol to compare copy ratios for each exon against an exon specific model of expected coverage and associated variance [GATK CA]. Those exons that are adjacent and significantly different are combined into larger CNV regions. This process was run for both the tumor and normal exome sample sets and filtered for those regions only present in the tumor (minimum absolute tumor log2 copy ratio >= 0.15, maximum absolute normal log2 copy ratio < 0.5, minimum absolute log2 tumor/normal copy ratio >= 0.15) using the USeq GatkCalledSegmentAnnotator. We next focused on the copy number calls in 11 genes of interest, specifically amplifications call in *AR, MYC, COL22A1, PIK3CA, PIK3CB and NOTCH1* and deletions call in *TMPRSS2, NCOR1, ZPTB18, TP53 and NKX3-1.* If MGple calls were identified for a given gene, the maximum log2 ratio was selected as the representative value within the amplified gene list (*AR, MYC, COL22A1, PIK3CA, PIK3CB and NOTCH1*). Conversely, for genes within the deletion gene list (*TMPRSS2, NCOR1, ZPTB18, TP53 and NKX3-1*), the minimum log2 copy ratio call was taken. Specifically, any deletion call detected in genes typically classified for amplification analysis (*AR, MYC, COL22A1, PIK3CA, PIK3CB*, or *NOTCH1*) was adjusted to a neutral status. Similarly, any amplification call detected in genes typically classified for deletion analysis (*TMPRSS2, NCOR1, ZPTB18, TP53,* or *NKX3-1*) was also adjusted to a neutral status, treating these amplifications as copy neutral. Next, a log2 copy ratio threshold of > 0.5 and <-0.5 was used to define genomic gain and genomic loss respectively.

***Plasma biopsy analysis of the PROMOTE cohort***

The copy number variation calls were directly extracted from our previously published work with the detailed method for copy number calls has been previously reported [2]. In brief, sequence reads from cfDNA sequencing data [6] were mapped to the human genome (hg19) using Bowtie-2 (Version 2.4.2) with the default settings [7]. SAMtools (Version 1.11) command lines were used to convert the file format from SAM to BAM, followed by sorting, indexing, and removing duplicate reads [8]. FeatureCounts from the Subread package (Release 2.0.3) was used to call read counts for each gene [9]. To make calls for the copy number status, a gene-specific log2 ratio was calculated by dividing sequence reads mapping to a gene in a patient to median sequence reads mapping to the same gene in normal controls, followed by log2 transformation. Genomic gain was defined as log2 ratio > 0.3 and genomic loss was defined as log2 ratio < -0.3.

***Extraction of Copy Number Variant (CNV) frequency from cBioPortal***

We searched cBioPortal (<https://www.cbioportal.org/>) for metastatic prostate cancer dataset. We identified five relevant datasets; (1) ‘Metastatic castration-sensitive prostate cancer” (424 samples) [10]; (2) “Metastatic Prostate Adenocarcinoma with mCRPC (121 samples) [11]; (3) “The Metastatic Prostate Cancer Project" (Provisional, June 2021) (123 samples); (4) “Metastatic Prostate Cancer (SU2C/PCF Dream Team, Cell 2015)” (150 samples) [12]; (5) “Metastatic Prostate Adenocarcinoma (SU2C/PCF Dream Team, PNAS 2019)” (444 samples) [3]. As we are interested in “treatment-naïve” mCRPC state (patients with no new drug exposure to mCRPC treatments), we identified a subset of 126 mCRPC samples that were treatment naive for Abiraterone, Enzalutamide and Taxane from the “Metastatic Prostate Adenocarcinoma” (SU2C/PCF Dream Team dataset) [3]. We next extracted the amplification (AMP) or deletion (HOMDEL) frequency of all the studies above by downloading the copy number alteration reports as provided on BioPortal summary page, specifically at the ‘CNA Genes’ report tab.

***MG-CNV risk score calculation***

***Selection of samples for risk score calculation***: In the PROMOTE treatment-naive-treatment cohort, 72 patients’ data were downloaded from dbGap. After removing samples with low sequencing coverage (as described above), 68 samples were retained. Similarly, in the PROMOTE post-treatment cohort, 75 patient data was available and after filtering, 74 samples were retained.

The SU2C/PCF Dream Team, PNAS 2019 cohort [3] contained 444 samples. To obtain individual patient CNV calls, we extracted the CNV data from the publicly provided link on the main study page, under the description "*Download all clinical and genomic data of this study*" (<https://cbioportal-datahub.s3.amazonaws.com/prad_su2c_2019.tar.gz>). CNV calls were binarized according to the PROMOTE data (detailed under section *Somatic Copy Ratio Analysis above*). Risk-score calculation was done in a subset of patients that were 1) Treatment naïve for Abiraterone, Enzalutamide and Taxane (n=163), 2) Classified as adenocarcinoma based on pathology (n=126), 3) Available copy number call in all 11 genes of interest (n=114), 4) Available clinical data on overall survival (n=96).

Vancouver Prostate Cancer and BC Cancer (“VPC” dataset) contained 382 samples [13]. Methods to call CNV from this cohort is detailed at <https://github.com/annalam/cfdna-wgs-manuscript-code>. As the dataset if from a targeted prostate-cancer relevant gene panel (~72 genes), only 8 genes (*AR, MYC, PIK3CA, PIK3CB, NKX3-1, TMPRSS2, TP53* and *ZBTB1*) out of the 11 genes of interest in our 11-gene CNV risk-score algorithm was available. Of the 382 samples, 335 contained copy number calls in all 8 genes. The CNV were binarized to 1 for gain, -1 for loss based on the log2 threshold of 0.3. Risk score calculation was performed on the set of 335 samples.

*Calculation of risk score values*: The MG-gene risk score calculation was adopted from our previous work [2]. We first calculated the Cox Regression coefficient (Hazard Ratio: HR) of each of the 11 genes by fitting a Cox proportional hazard regression model to clinical variables (as described above). Next, to obtain a MG-gene risk score for an individual patient, we implemented the following risk score formula: Sum [Cox regression coefficient × CNV status (1 for gain or loss, and 0 for no change) of each gene. Finally, we dichotomized the risk score on its median value for all patients, with a risk score above the median labelled “high-risk” and below the median as “low-risk”.

***Calculation of Cox regression coefficient methods and survival analysis***

Cox proportional regression analysis was conducted for each of the 11 genes of interest using the *survival* package (version 3.4-0). For each gene, the CNV status was binarized to 1 or 0, with 1 representing gain or loss and 0 representing no change. The CNV status was subsequently treated as the independent variable while the Progression-Free Survival (PFS) or Overall Survival (OS) were treated as the independent (outcome) variable. In the Vancouver datasets [13], univariate and MGvariate cox regression analysis was also conducted on other outcome variable including hemoglobin (Hb), Lactate Dehydrogenase (LDH) and Alkaline phosphatase (ALP). Kaplan-Meier survival curves were generated using the *survival* package with the function *Surv*. Survival plots were generated with the *survminer* package (version 0.4.9). All analysis was conducted in R (version 4.2.2). Analysis scripts are available at <https://github.com/zakiF/PublishedPapers/ProstatePROMOTE>.

***Copy number Concordance between plasma and metastatic tissue CNVs***

To evaluate the agreement of copy number calls within the PROMOTE cohort, we compared copy number calls between metastatic tissue samples and plasma biopsies obtained from the same set of patients. To match the publicly available plasma biopsy PROMOTE data, we binarized the metastatic tissue CNV calls to 1/-1 for gain or loss respectively, and 0 for no change. We visualized the copy CNV using the oncoPrint function from the R (Version 4.2.2) package ‘ComplexHeatmap’ (Version 2.15.1). To determine concordance, we created a 2x2 contingency table from which the Cohen’s Kappa (κ), positive predictive value (PPV), negative predictive value (NPV) and prevalence was calculated. Prior to generation of the 2x2 contingency table, all CNV calls (gain or deletion) was converted to 1 and copy neutral was converted to 0. From a given contingency table, the following formula were used to calculate the above-mentioned statistics:

|  |  | Metastatic tissue CNV call | |  |
| --- | --- | --- | --- | --- |
|  |  |  |  |  |
|  |  | **Yes** | **No** | **TOTAL** |
| Plasma CNV  call | **Yes** | TP | FP | TP + FP |
|  | **No** | FN | TN | FN + TN |
|  | **TOTAL** | TP + FN | FP + TN | **N** |

- TP (true positive) = Metastatic tissue positive and Plasma positive
- FP (false positive) = Metastatic tissue negative and Plasma positive
- FN (false negative) = Metastatic tissue positive and Plasma negative
- TN (true negative) = Metastatic tissue negative and Plasma negative
- N = Total number of CNV calls
- Positive Predictive Value (PPV) = 100xTP/(TP+FP)
- Negative Predictive Value (NPV) = 100xTN/(FN+TN)
- Prevalence = 100x(TP+FN)/N

References

1. Sicotte, H., et al., *Molecular Profile Changes in Patients with Castrate-Resistant Prostate Cancer Pre- and Post-Abiraterone/Prednisone Treatment.* Mol Cancer Res, 2022. **20**(12): p. 1739-1750.

2. Huang, J., et al., *Plasma Copy Number Alteration-Based Prognostic and Predictive Multi-Gene Risk Score in Metastatic Castration-Resistant Prostate Cancer.* Cancers (Basel), 2022. **14**(19).

3. Abida, W., et al., *Genomic correlates of clinical outcome in advanced prostate cancer.* Proc Natl Acad Sci U S A, 2019. **116**(23): p. 11428-11436.

4. Wang, L., et al., *A prospective genome-wide study of prostate cancer metastases reveals association of wnt pathway activation and increased cell cycle proliferation with primary resistance to abiraterone acetate-prednisone.* Ann Oncol, 2018. **29**(2): p. 352-360.

5. Scher, H.I., et al., *Design and end points of clinical trials for patients with progressive prostate cancer and castrate levels of testosterone: recommendations of the Prostate Cancer Clinical Trials Working Group.* Journal of clinical oncology : official journal of the American Society of Clinical Oncology, 2008. **26**(7): p. 1148-59.

6. Du, M., et al., *Plasma cell-free DNA-based predictors of response to abiraterone acetate/prednisone and prognostic factors in metastatic castration-resistant prostate cancer.* Prostate Cancer Prostatic Dis, 2020. **23**(4): p. 705-713.

7. Langmead, B. and S.L. Salzberg, *Fast gapped-read alignment with Bowtie 2.* Nat Methods, 2012. **9**(4): p. 357-9.

8. Li, H., et al., *The Sequence Alignment/Map format and SAMtools.* Bioinformatics, 2009. **25**(16): p. 2078-9.

9. Liao, Y., G.K. Smyth, and W. Shi, *featureCounts: an efficient general purpose program for assigning sequence reads to genomic features.* Bioinformatics, 2014. **30**(7): p. 923-30.

10. Stopsack, K.H., et al., *Oncogenic Genomic Alterations, Clinical Phenotypes, and Outcomes in Metastatic Castration-Sensitive Prostate Cancer.* Clin Cancer Res, 2020. **26**(13): p. 3230-3238.

11. Grasso, C.S., et al., *The mutational landscape of lethal castration-resistant prostate cancer.* Nature, 2012. **487**(7406): p. 239-43.

12. Robinson, D., et al., *Integrative clinical genomics of advanced prostate cancer.* Cell, 2015. **161**(5): p. 1215-28.

13. Fonseca, N.M., et al., *Prediction of plasma ctDNA fraction and prognostic implications of liquid biopsy in advanced prostate cancer.* Nat Commun, 2024. **15**(1): p. 1828.

# **Supplementary Results**

**Supplementary Data Results:**

*Description of the dbGaP “PROMOTE” matched tissue-plasma study cohort with final biospecimen numbers for study analysis*

De-identified publicly accessed genomic and clinical data for the “PROMOTE” cohort ^1,2^ includes concurrently collected matched tissue-plasma ^3^ pairs at a “treatment-naive” and a second “post-treatment” time point after 12 weeks of abiraterone-acetate/prednisone (AA/P) therapy in the first-line mCRPC clinical setting. Sequencing details linking the de-identified tissue and cfDNA pair IDs alongside the clinical information are provided in “**Supplementary Table 2**”. **Figure 1A** illustrates the study schema and the number of matched plasma-metastatic biopsy pairs available in each of the two serial visits accessed for analysis (N=311), along with the number of metastatic tissue biopsies performed in bone versus non-bone sites in each serial visits. Of the 82 cfDNA biospecimen with sequencing data available ^3^ there were 72 matched metastatic tissue biospecimen pairs with sequencing data available for analysis at the treatment-naive time-point in dbGaP. At the 12-week post AA/P treatment time-point of the 82-post treatment cfDNA biospecimen, 75 matched metastatic tissue biospecimen sequencing data were available in dbGaP (**Figure 1A**). Of the 72 treatment-naive and 75 post-treatment metastatic solid tissue samples, 4/72 and 1/75 were excluded from further analysis due to poor sequencing coverage **Figure 1A** also details the metastatic biopsy sites for the 72-treatment-naive metastatic tissue biospecimen sequencing data include 48/72 from bone and 17/72 from non-bone sites. In the 75 post-treatment sequencing data, 62/75 were performed in bone tissue and 13/75 in non-bone metastatic tissue. After passing QC in the PROMOTE cohort, analysis is reported for 68 treatment-naive and 74 post-treatment biopsies matched metastatic-tissue and cfDNA biospecimen pairs (**Supplementary Figure 1A**).

*Copy number variation (CNV) call thresholds in the PROMOTE metastatic tissue biospecimen*

A study goal was to determine the performance of a 11-gene CNV cfDNA risk-score previously reported in the PROMOTE dataset ^3^ in metastatic tissue from PROMOTE and cBioPortal and in the VPC plasma cfDNA cohorts to predict OS and PFS. These 11 gene CNVs include gains in *AR, MYC, COL22A1, PIK3CA, PIK3CB and NOTCH1* and loss in *TMPRSS2, NCOR1, ZBTB16, TP53 and NKX3-1.* We first established thresholds for CNV calls using the PROMOTE dbGaP metastatic tissue sequencing results. We observed that thresholds to call copy number gain or loss from WES data varies in reported literature, from a lenient log2 ratio of 0.1 ^4^ to a more stringent log2 ratio of 0.4 ^5^. Therefore, we determined an optimal threshold for calling CNVs in the 11 genes of interest by evaluating thresholds ranging from 0.1 to 0.5 log2 ratios. Using the set of 68 treatment-naive tissue samples, we identified CNV calls for each threshold and calculated the corresponding CNV frequencies. We then compared these CNV frequencies with the reported CNV frequencies for the same 11 genes in six independent public mCRPC datasets available in cBioPortal (**Supplementary Figure 2**) for establishing the most optimal log2 ratio that produces comparable CNV frequencies across all datasets. We observed that the lenient log2 ratio threshold for CNV calls in the PROMOTE database specimens increased the frequency of individual gene CNV calls, suggesting that thresholds for CNV calls are an important parameter to consider. In general, applying an absolute log2 ratio of 0.5 to PROMOTE generated comparable frequency to the public datasets for several CNV genes. For example, the frequency of *NOTCH1* amplification in the SU2C/PCF cBioPortal dataset was 4%, similar to that observed in the PROMOTE dataset (4%) and in the SU2C/PCF Dream Team “Treatment-Naïve” mCRPC sub-cohort dataset ^6^ at 5%. The comparative frequencies for all CNV gains and loss are detailed in **Supplementary Figure 2.** Based on these observations, the most optimal log2 ratio cut-off for gains and loss was deemed at log2 ratio ±0.5. We have detailed the CNV calls for all PROMOTE tissue biospecimen for both visits in **Supplementary Table 3and**  utilizing the log2 ratio of ±0.5 to call gains/loss, we binarized the CNV calls as 1 for gain, -1 for loss and 0 for no change.

*Tissue DNA tumor purity impact on CNV frequency in metastatic tissue and plasma biospecimen*

Since the majority of the PROMOTE solid tissue metastases biopsies were bone-based (**Figure 1A**) which is a common metastatic route for prostate cancer spread, and skeletal biopsies can yield low DNA purity ^7^ which may interfere with CNV calls, we considered the impact of tumor DNA purity on CNV detection in the 11 candidate CNVs of interest. **Supplementary Table 2** lists the individual tumor DNA purity for the metastatic tissue biospecimen along with the tumor fraction (also called as the circulating tumor, ctDNA) of the matched plasma biospecimen for both serial visits. We set the tissue tumor DNA purity threshold at <20% tumor DNA purity, to be labelled as “low” and >/=20% as “high” in line with previous reports published in the SU2C/PCF dataset samples ^8^. Metastatic tissues with low tumor DNA purity were more frequently observed in the bone site specimens (N=55/110, 50%) compared to non-bone tissue site specimens (N=5/32, 16%) (**Figure 2A**) (P=5.2 x10^-5^). We also observed a significant positive correlation for the number of CNV calls and tumor DNA purity for both bone and non-bone sites (**Figure 2B**). This correlation was stronger for metastatic bone tissues (R=0.78) than for non-bone metastatic tissue sites (R=0.66) (**Figure 2B**). Similarly, we were also interested in determining the effect of ctDNA in the matched plasma biospecimen on CNV calls and their correlation. The distribution of ctDNA in all plasma biospecimen is shown in **Figure 2C.** A positive correlation of CNV calls with tumor fraction was also observed with the number of CNV calls increasing with tumor fraction (R=0.75) (**Figure 2D**). Based on the distribution of the ctDNA tumor fraction in **Figure 2C**, this cfDNA biospecimen set was characterized as high versus low tumor fraction at a cut-off of greater than or less than the third quartile in the distribution of the cohort, which corresponded to ctDNA </> 6.47%.

*Landscape of CNV calls in metastatic “gold-standard” tissue and matched cfDNA biospecimen in PROMOTE dataset for treatment-naïve and 12-week post-treatment time points*

The landscape of CNV tissue-based calls for all 11 CNVs in the 68 treatment-naive samples matched with the CNVs reported from concurrently matched cfDNA specimens is shown in **Figure 3A.** The CNV landscape is presented based on the site of metastatic tissue and on “high” and “low” tumor tissue DNA purity**.** As only 3/20 non-bone treatment-naive biospecimen had low tumor DNA purity, we did not distinguish non-bone samples into “high” or “low” tumor purity groups for performing study analyses and these non-bone site group was analyzed together (n=20), while the bone-based site was grouped and analyzed based on high (n=28/48) and low (n=20/48) tissue tumor DNA purity.

Across all the tissue samples and regardless of tumor DNA purity or metastatic site, *AR* gain and *NKX3-1* loss in tissue were the most frequently altered genes in both tissue and plasma cfDNA biopsy biospecimen (**Figure 3B**). CNV calls in solid tissue bone biopsies with low tumor DNA purity were absent, except for *AR* gain detected in 20% of tissues samples (**Figure 3B**). The degree of agreement for CNV calls between matched treatment-naive tissue and cfDNA pairs with high tumor purity and high tumor fraction were measured using a *Kappa-statistic*. In the bone-based biopsy with high tumor purity, 10 biospecimen were also identified as having high tumor fractions (**Figure 3A**). The tissue and cfDNA pairs agreement of CNV calls in these 10 samples is listed as a contingency table (**Figure 3C**). We observed a *Kappa* of 0.33 between plasma and solid tissue biopsies for detecting CNVs in the group of high tumor DNA purity bone-based biopsies and high tumor fraction suggesting a fair level of agreement between the two biopsy approaches when both the tissue and plasma cfDNA yields are high. Based on the prevalence (of CNVs) in the contingency table, the positive and negative predictive values (PPV/NPV) of plasma cfDNA-based biopsy detected CNVs against the gold standard reference of solid tissue biopsy CNV was calculated. In this group of patients with high tumor DNA purity and high tumor fraction, the PPV for cfDNA biopsies was 46.2% and the NPV for plasma biopsies was 82.2%.

The landscape of CNV calls in 74 metastatic tissue and matched cfDNA pairs for all 11 CNVs at the 12-week post-treatment time-point for each individual patient are shown in **Supplementary Figure 3.**

*MG-CNV risk score and clinical outcomes in the matched tissue biospecimen*

Our study goal was to determine the performance of the previously reported MG-CNV risk score in plasma cfDNA-based biospecimen ^3^ to predict clinical outcomes in matched metastatic tissue biospecimen. Calculation of the risk score using tissue based CNVs and definition of “high” risk and “low” risk scores is provided in “**Supplementary Methods”**. **Supplementary Table 4** lists individual patient risk scores generated for biospecimen from treatment-naive and post 12-week treatments visits for the clinical outcomes of overall survival (OS) and progression free survival (PFS) for AA/P therapy. Clinical response to AA/P therapy of each research subject at 12-weeks is also listed. The range of the calculated tissue-based risk scores for predicting OS in the treatment-naive visit was observed to be between -0.11 to 4.01 with a median of 0.37 and 30/68 patients having high-risk score values (above the median) and with a median survival of 24.9 months (Range: 3.7–47.5 months) compared to patients with low-risk score (n=38/68) with a median survival of 30.6 months (Range: 7.9–51.8) months (P=0.039). The range of the calculated risk score for predicting PFS was between -1.16 to 3.14, (median 0.25). Patients with high-risk scores showed a median PFS of 7.8 months compared to 14 months in patients with low-risk score. The *Kaplan-Meier* survival plots for OS and PFS respectively in treatment-naïve mCRPC patients based on the tissue MG-CNV risk score (high Vs. low) are shown in **Figure 4A** and **Figure 4B**. To determine if the metastatic site and DNA tumor purity were also predictive of OS/PFS, we explored the impact of metastatic site biopsy (bone versus non-bone) and high versus low DNA tumor purity to predict OS/PFS and found no impact of these factors on clinical outcomes (**Supplementary Figure 4**).

*Pharmacodynamic changes in the MG-CNV tissue-based risk score after 12-weeks of abiraterone acetate/prednisone therapy*

As this study cohort had prospectively collected serial tissue and plasma biospecimens at treatment-naive and after 12-weeks of AA/P treatments (**Figure 1A**), we evaluated the pharmacodynamic impact of 12-week AA/P drug treatment on the tissue-derived MG-CNV risk score. Results of all research subjects’ 12-week response to treatment using a composite progression free survival evaluation criterion for assessment as detailed in **Supplementary Methods,** are listed in **Supplementary Table 4**. Of the 68 research subject, 60 had matched serial pre/post 12-week treatment tissue biopsy sequencing data and clinical response available for analysis and 31/60 were listed as responders at 12-weeks and 29/60 non-responders at 12 weeks. **Figure 4C** demonstrates changes between pre and 12-week post treatment risk score for each individual patient based on treatment response at 12-weeks. Patients with primary resistance to AA/P (non-responder group at 12-weeks) were observed to have a significant increase in the risk score.

*Impact of biopsy site and treatment-naive tumor purity on survival*

In the matched metastatic tissue from bone biopsy sites, we observed lower tumor tissue DNA purity (**Figure 2A**) in bone biospecimen, but also observed that regardless of the site biopsied the overall CNV detection frequency correlates with tissue DNA purity (**Figure 2B**). Thus, for descriptive clarity on the role of biopsy site and tissue DNA purity we grouped our analysis of the metastatic tissue biospecimen CNV calls based on DNA purity and site of metastases. Of note, the mere presence of site biopsied (bone versus non-bone) or tissue DNA purity alone was not associated with survival or progression free survival outcomes (**Supplementary Figures 4 A-D**), but the collective score generated from presence or absence of specific CNV alterations discriminates survival outcomes (**Figure 4A, 4B**). These observations may suggest the importance of specific tumor biology related alterations to predict clinical outcomes is more relevant that biopsy site tissue purity as our results for the MG-CNV score to associate with survival outcome were positive in not only this tissue dataset, but also in the independent cBioPortal tissue dataset.

*Concordance in the PROMOTE matched tissue-plasma biopsy pairs*

We observed that the *Kappa*-statistic, a measure used for assessing inter-observational reliability of agreement was 0.33 (**Figure 2C**), which indicates a fair agreement in the matched tissue-plasma pairs. The resulting positive and negative predictive values (PPV/NPV) for plasma biospecimen calls are 45.9% and 84.9% when comparing plasma biospecimen with high ctDNA to matched tissue biospecimen with high tissue purity. We were also able to observe that there are low CNV frequencies for the 11 individual genes in bone biospecimens with low tumor DNA purity, in both pre and 12-week post treatment collections (**Figure 3B and Supplementary Figure 3B**), which will impact PPV/NPV percentages when understanding predictive values of plasma-based biospecimen.

*Results in metastatic tissue-based treatment-naïve mCRPC SU2C/PCF cBioPortal cohort biospecimen dataset*

Sequencing data accessed of mCRPC patients (N=444) ^6^ in cBioPortal which also had clinical outcomes was accessed. This dataset of 444 patients included 163 treatment-naive mCRPC patients who had metastatic tissue biopsies performed prior to initiation of first-line treatment with abiraterone acetate/prednisone or enzalutamide. Of these 96 treatment-naive patients had survival data publicly available (**Supplementary Figure 1B**), which were analyzed for MG-CNV score performance.

*MG-CNV risk score performance in independent metastatic tissue-based treatment-naïve mCRPC SU2C/PCF cBioPortal dataset*

The MG-CNV risk score was evaluated in an independent cohort of treatment-naïve mCRPC patients for predicting overall survival (**Figure 1B**). Sequencing data accessed of mCRPC patients (N=444) ^6^ in cBioPortal which also had clinical outcomes was accessed. This dataset of 444 patients included 163 treatment-naive mCRPC patients who had metastatic tissue biopsies performed prior to initiation of first-line treatment with abiraterone acetate/prednisone or enzalutamide, of which 96 treatment-naive patients had survival data available (**Supplementary Figure 1B**). We applied the CNV call criteria as for the “PROMOTE” CNV dataset of 11 CNVs to this cohort and calculated the MG-CNV risk score for all 96 biospecimens. The risk-score ranged from -0.45 to 3.59, with a median of 1.94 (**Supplementary Table 5**) with 46/92 patients classified in the “high-risk” category (above the median) and 46/92 as low-risk patients. We observed that patients with high-risk score had a lower (median) survival of 22.2 months compared to patients with low-risk score at 33.7 months (**Figure 5A**).

This cohort included two integrative gene transcription scores for predicting clinical outcomes, an “*AR score*” and a “*NEPC score*” ^6,9^ which have been reported previously to potentially indicate aggressive clinical behavior in mCRPC state ^1,2^. **Supplementary Table 5** lists the individual *AR* and NEPC scores for the treatment-naïve biospecimens that were previously reported in the treatment-naive tissue biospecimen set ^1^. The scores were dichotomized at the median for the respective ranges into “high” and “low”. We evaluated these scores for predicting survival after dichotomizing into “high” and “low” based on above and below the median for the ranges provided. No association with overall survival for either score was observed (**Supplementary Figure 5**).

*MG-CNV-based risk score performance in the Vancouver Prostate Cancer (VPC) plasma biopsy-based mCRPC cohort*

We also evaluated the MG-CNV risk score performance in the VPC cohort of 382 treatment-naïve mCRPC patients (**Figure 1C**). Patients in this cohort had undergone plasma biopsy profiling for somatic alterations using a hybrid-capture panel of 72 genes for predicting mCRPC treatment and survival outcomes. The hybrid-capture panel included CNV calls for 8 out of the 11 genes of interest in our 11-gene CNV risk-score algorithm, specifically: *AR, MYC, PIK3CA, PIK3CB, NKX3-1, TMPRSS2, TP53* and *ZBTB16*. A subset of these patients (N=335/382) contained CNV data for all 8 genes of interest. In this cohort, the risk-score range for predicting OS were between 0 to 8.94 (median=in. Similar to previous risk-score stratification, patients were dichotomized into “high-risk” or “low-risk” groups based on the risk-score value above or below the respective medians. For overall survival endpoint, of the 335 patients 135 patients were classified as “high-risk” with a median survival of 15.5 months compared to the 200 patients classified as “low-risk” with a median survival of 38.8 months (**Figure 4B**). **Supplementary Table 6** lists individual patient MG-CNV score (as “high-risk” or low-risk” category, lab-values, subsequent treatments, PSA-progression free survival (PSA-PFS) and overall survival (OS) for all patients in this cohort.

To evaluate the risk-score performance as a predictor of clinical outcome, we analyzed the hazard ratio of the MG-CNV risk score and compared it with the hazard ratios of other clinical prognostic factors for mCRPC state including hemoglobin (Hgb), Lactate Dehydrogenase (LDH) and Alkaline phosphatase (ALP) levels at the univariate and MGvariate Cox regression level. We observed that all the clinical factors and the MG-CNV risk score had significant Hazard Ratios (HRs) for predicting overall survival at the univariate level **Supplementary Table 7**. At the MG-variate level, the highest HR for survival was observed for the MG-CNV risk score (HR: 2.64; range 1.88-3.51; p=1.35 x10^-11^) (**Figure 5C** and **Supplementary Table 7).**

Finally, we investigated the 8-gene MG-CNV risk-score to predict treatment response to AA/P and enzalutamide as defined by PSA-based progression free survival (PSA-PFS). In the VPC cohort, 137 patients were treated with AA/P and 174 patients were treated with enzalutamide. Of the 137 patients subsequently treated with AA/P, based on the calculated risk score 51 patients were classified as “high-risk” and 86/137 were “low-risk”. For the 174 patients treated with Enzalutamide 71/174 were classified as “high-risk” and 102 were in the “low-risk” group. The median PSA-PFS for AA/P in the “high-risk” group was 5.5 months and 11.7 months in the “low-risk” group. The median PSA PFS for the “high-risk” group of patients subsequently treated with enzalutamide was 5.4 months and 14.2 months for the “low-risk” group. **Figure 5D** illustrates the survival curves for both treatments based on risk scores and PSA-PFS endpoint.

References

1. Wang L, Dehm SM, Hillman DW, et al. A prospective genome-wide study of prostate cancer metastases reveals association of wnt pathway activation and increased cell cycle proliferation with primary resistance to abiraterone acetate-prednisone. *Ann Oncol*. Feb 1 2018;29(2):352-360. doi:10.1093/annonc/mdx689

2. Sicotte H, Kalari KR, Qin S, et al. Molecular Profile Changes in Patients with Castrate-Resistant Prostate Cancer Pre- and Post-Abiraterone/Prednisone Treatment. *Mol Cancer Res*. Dec 2 2022;20(12):1739-1750. doi:10.1158/1541-7786.MCR-22-0099

3. Huang J, Du M, Soupir A, et al. Plasma Copy Number Alteration-Based Prognostic and Predictive Multi-Gene Risk Score in Metastatic Castration-Resistant Prostate Cancer. *Cancers (Basel)*. Sep 28 2022;14(19)doi:10.3390/cancers14194714

4. Wyatt AW, Annala M, Aggarwal R, et al. Concordance of Circulating Tumor DNA and Matched Metastatic Tissue Biopsy in Prostate Cancer. *J Natl Cancer Inst*. Dec 1 2017;109(12)doi:10.1093/jnci/djx118

5. de Ligt J, Boone PM, Pfundt R, et al. Detection of clinically relevant copy number variants with whole-exome sequencing. *Hum Mutat*. Oct 2013;34(10):1439-48. doi:10.1002/humu.22387

6. Abida W, Cyrta J, Heller G, et al. Genomic correlates of clinical outcome in advanced prostate cancer. *Proc Natl Acad Sci U S A*. Jun 4 2019;116(23):11428-11436. doi:10.1073/pnas.1902651116

7. Jimenez RE, Atwell TD, Sicotte H, et al. A Prospective Correlation of Tissue Histopathology With Nucleic Acid Yield in Metastatic Castration-Resistant Prostate Cancer Biopsy Specimens. *Mayo Clin Proc Innov Qual Outcomes*. Mar 2019;3(1):14-22. doi:10.1016/j.mayocpiqo.2018.12.005

8. Robinson D, Van Allen EM, Wu YM, et al. Integrative clinical genomics of advanced prostate cancer. *Cell*. May 21 2015;161(5):1215-28. doi:10.1016/j.cell.2015.05.001

9. Beltran H, Prandi D, Mosquera JM, et al. Divergent clonal evolution of castration-resistant neuroendocrine prostate cancer. *Nat Med*. Mar 2016;22(3):298-305. doi:10.1038/nm.4045

# **Supplementary Tables Legend**

**Supplementary Table 1**: List of recent concordance studies in advanced prostate cancer that have compared metastatic tissue and cfDNA-based landscapes of molecular alterations

**Supplementary Table 2**: Metadata of the PROMOTE cohort. De-identified solid tissue biospecimen from whole exome sequencing files extracted from PROMOTE dbGaP database with IDs matched across dbGaP database [1] and the PROMOTE liquid (plasma) biopsy database [2] for the same patient for treatment-naive and 12-week post treatment visits for all biospecimens. Clinical outcomes of all patients listed as reported at the time of study cutoff date of follow up include overall survival, progression free survival and 12-week response to AA/P.

**Supplementary Table 3**: Log2 ratio of solid tissue copy number variant calls at treatment-naive and 12-week post-treatment for the PROMOTE biospecimens across 11 genes of interest matched to the PROMOTE liquid (plasma) biospecimen IDs [1]. Copy number calls are provided as log2 ratio values and binarized to 1 for gain, -1 for loss and 0 for no change. Thresholds for CNV calls used to binarize gain or loss is **set at log2 ratio of ±** 0.5.

**Supplementary Table 4**: PROMOTE tissue biospecimen multi-CNV (11 genes) risk score values calculated prior to initiating abiraterone acetate/prednisone-treatment-naive and 12-week post treatment time points. The multi-CNV risk score values are stratified to high risk (above median) and low risk (below median) for both individual time points and for overall survival and progression free survival. Progression on drug therapy after initial 12-week treatments is also listed for each subject.

**Supplementary Table 5**: Copy number calls extracted from treatment-naïve mCRPC cBioPortal SU2C/PCF cohort study [3] along with database IDs and individual patient clinical outcomes death and overall survival. The calculated risk score values are stratified to high risk (above median) and low risk (below median). 1; gene with copy number change, 0; no change. Also provided are the AR score and the NEPC score values, which were reported for each research subject and are listed for each subject either as “high” or “low” based on above or below the median calculated from the range of the individual score values

**Supplementary Table 6:**Treatment-naïve mCRPC cohort of liquid (plasma) biopsies(N=325) from the VPC cohort at University of British Columbia with CNV information on 8 genes (*AR, MYC, PIK3CA, PIK3CB, NKX3.1, TP53, TMPRSS, ZBTB16*), which are common to the set of 11 gene CNVs from other databases included in this study. Clinical outcomes of each research subject are also listed and include Overall survival, PSA-Progression Free Survival (PFS), type of first-line systemic treatments and risk category of the multi-CNV score calculated for each research subject as “high” versus “low” risk score for OS and PSA-PFS

**Supplementary Table 7**: Cox Proportional Hazards analysis for Overall survival in the VPC Cohort at University of British Columbia liquid (plasma) biopsy cohort at the univariate and multivariate level after adjusting for clinical laboratory prognostic factors. (ALP- serum Alkaline Phosphatase; HgB- Hemoglobin; LDH- Serum Lactate Dehydrogenase)

**References**

1. Sicotte, H., et al., *Molecular Profile Changes in Patients with Castrate-Resistant Prostate Cancer Pre- and Post-Abiraterone/Prednisone Treatment.* Mol Cancer Res, 2022. **20**(12): p. 1739-1750.

2. Huang, J., et al., *Plasma Copy Number Alteration-Based Prognostic and Predictive Multi-Gene Risk Score in Metastatic Castration-Resistant Prostate Cancer.* Cancers (Basel), 2022. **14**(19).

3. Abida, W., et al., *Genomic correlates of clinical outcome in advanced prostate cancer.* Proc Natl Acad Sci U S A, 2019. **116**(23): p. 11428-11436.

# **Supplementary Figures**

## **Supplementary Figure 1: Patient biospecimen filtering in all cohorts**

**
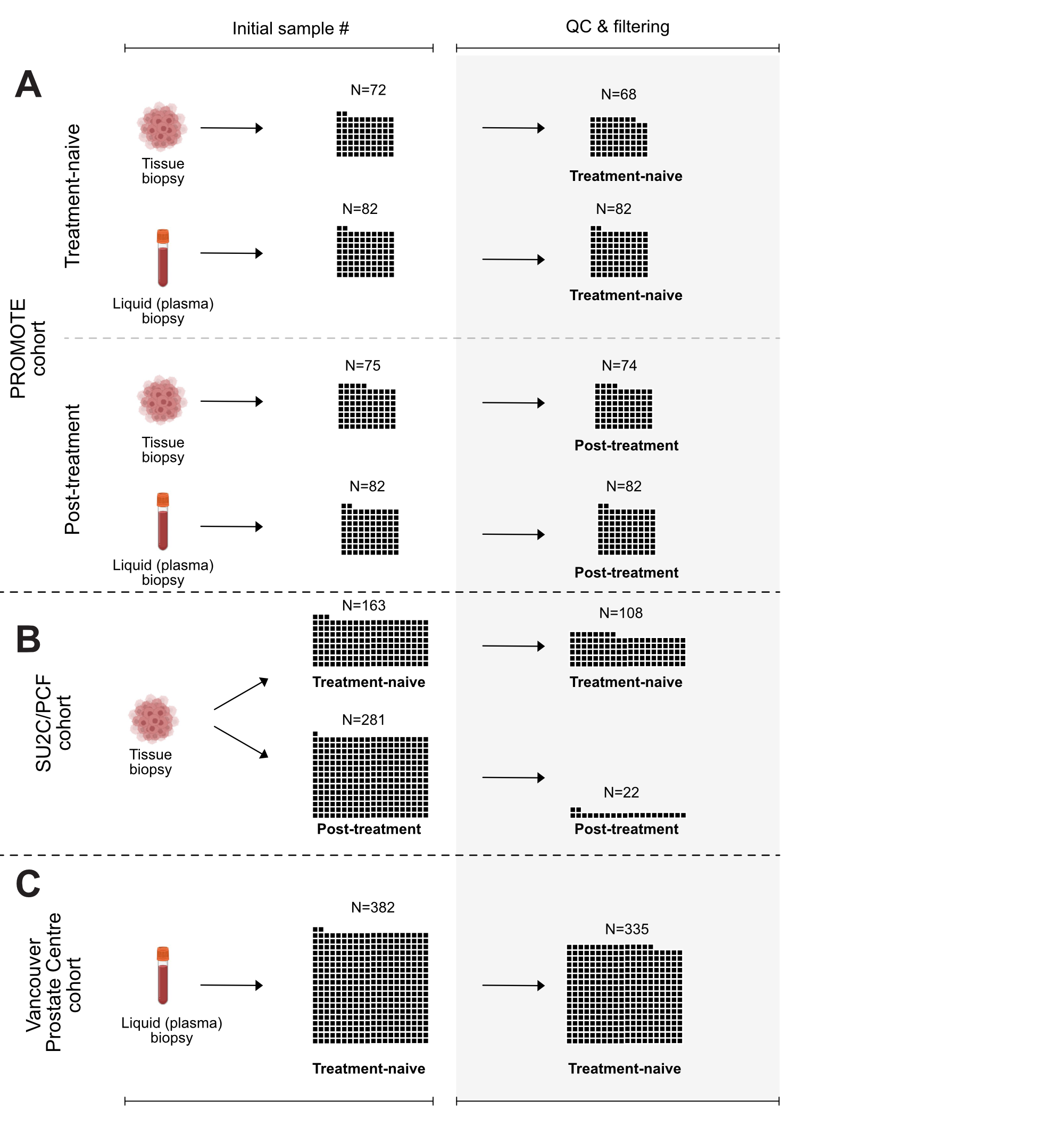
Supplementary Figure 1: Patient biospecimen filtering in all cohorts**

**S1A:** The initial number of PROMOTE biospecimen downloaded from dbGaP are shown (left). Tissue biospecimen whole exome sequencing data were analyzed and filtered to select specimens with sufficient sequencing coverage depth (right). Similarly, the number of concurrently collected liquid (plasma) biospecimen from the PORMOTE cohort were shown (left). All plasma samples had passed QC (right).

**S1B:** Tissue samples available in the SU2C/PCF cohort were extracted from cBioPortal (left). SU2C/PCF contained treatment-naïve and post-treatment samples. Samples were filtered to those with clinical outcome information and CNV calls in all 11-genes of interest (right).

**S1C:** The Vancouver Prostate Cancer consists of all treatment-naive liquid (plasma) patient (left). Samples were filtered to those with clinical outcome information and CNV calls in all 11-genes of interest (right).

## **Supplementary Figure 2: Frequency of Copy Number Variations (CNVs) in metastatic tissue datasets for 11 genes of interest**


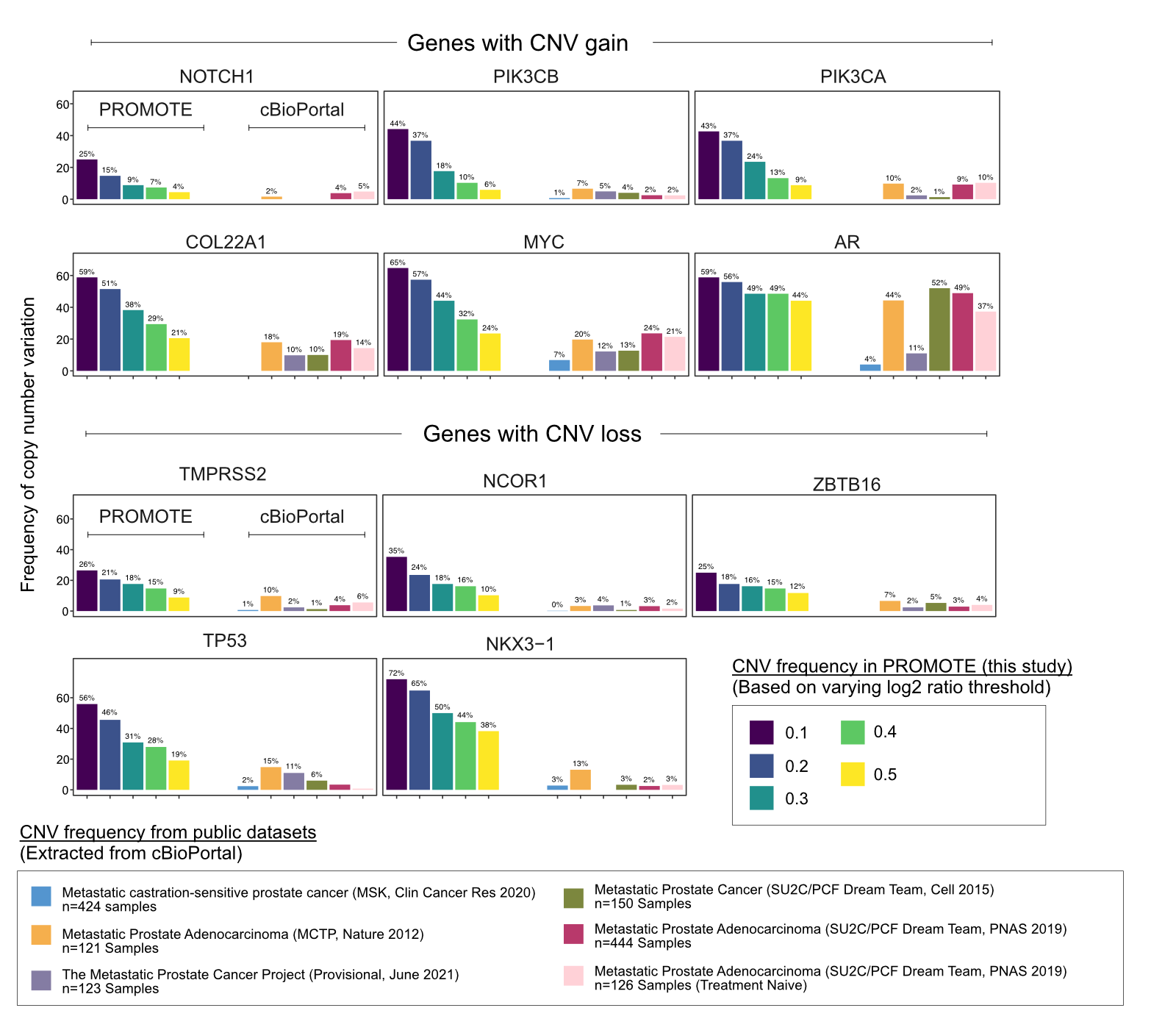


**Supplementary Figure 2: Frequency of Copy Number Variations (CNVs) in metastatic tissue datasets for 11 genes of interest**

The frequency of CNV gains (top) and CNV losses (bottom) for the 11 genes are shown across six metastatic tissue datasets listed in different colored bars. Two sets of bar graphs are represented for each gene plot. The bar plots on the left for each gene represents the frequency of CNV in the treatment-naive PROMOTE dataset at different log2 ratio threshold, ranging from 0.1 (lenient) to 0.5 (stringent). The bar plots on the right for each gene represents the frequency of CNVs observed for the six metastatic tissue cBioPortal datasets for the corresponding log2 ratio threshold.

## **Supplementary Figure 3: Landscape of Copy Number Variations (CNV)s in the 12-week post treatment biospecimen dataset from PROMOTE**

**
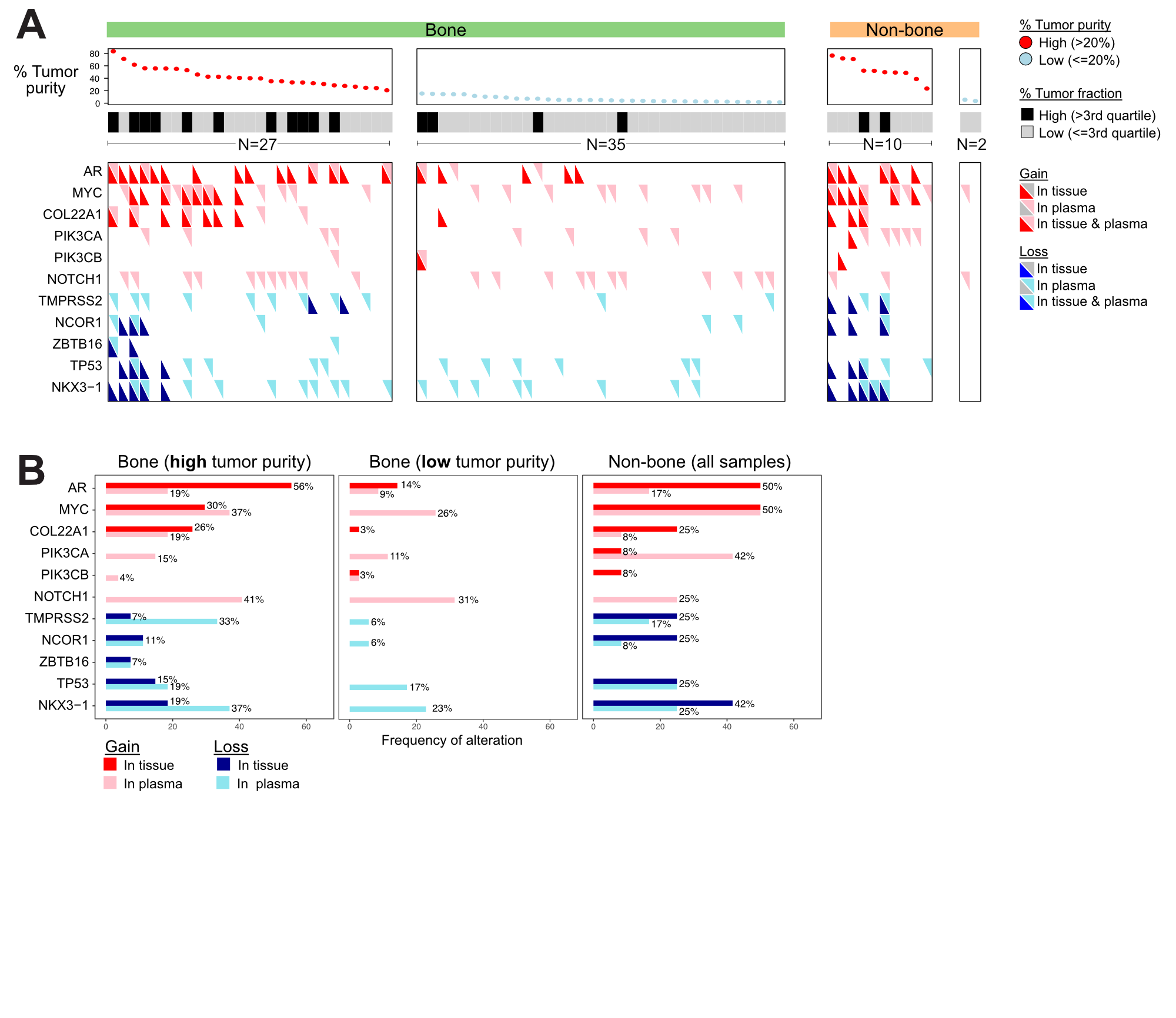
**

**Supplementary Figure 3: Landscape of Copy Number Variations (CNV)s in the 12-week post treatment biospecimen dataset from PROMOTE**

**S3A:** Heatmap showing individual patients in columns and the CNV calls for the 11-genes in rows in the matched tissue-plasma post-treatment biopsies. CNV calls in the tumor tissue is represented by red triangles for CNV gains and blue triangles for CNV loss. Matching CNV calls in liquid (plasma) biopsy is represented by light pink triangles for gains and light blue triangles for CNV loss. Patients are grouped based on tissue biopsies performed in bone and non-bone sites and on the basis of DNA tumor purity in metastatic tissue (<= or > 20%). The categorization of matched plasma ctDNA fraction to high- or low- tumor fraction is based on the 3^rd^ quartile range (ctDNA = 6.47%).

**S3B:** Frequency of CNVs detected in paired tissue (red/blue bars) and plasma (faint pink/blue bars) for the 11 genes of interest with red bars for CNV gains and blue for CNV loss. Frequency is grouped based on metastatic sites and DNA tumor purity.

## **Supplementary Figure 4: Association of metastatic tissue tumor site and DNA tumor purity with overall survival (OS) and progression free survival (PFS) in treatment-naive PROMOTE tissue biospecimen**

**
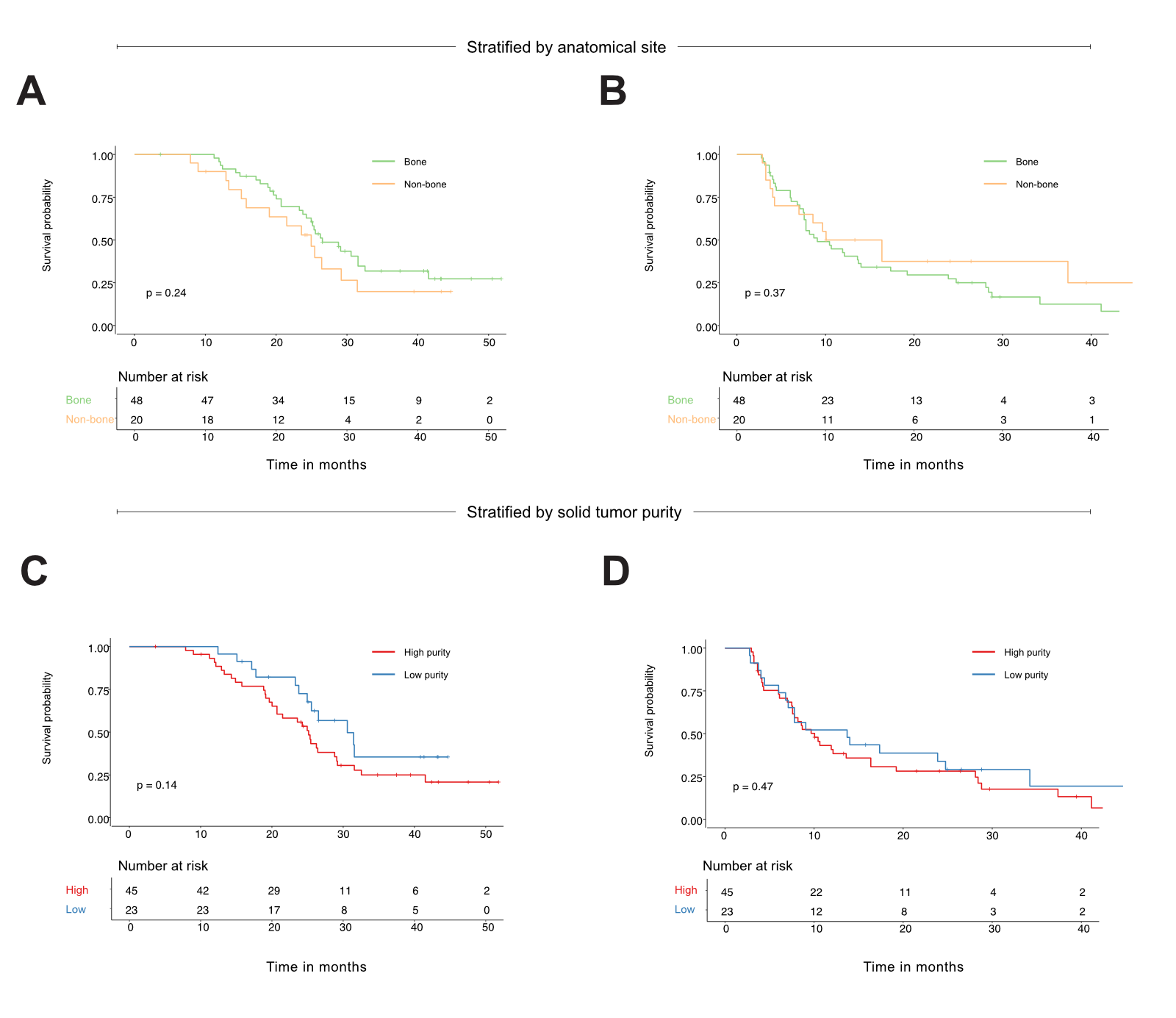
**

**Supplementary Figure 4: Association of metastatic tissue tumor site and DNA tumor purity with overall survival (OS) and progression free survival (PFS) in treatment-naive PROMOTE tissue biospecimen**

**S4A-B**: Kaplan-Meier survival plots stratified by anatomical site, between bone and non-bone tissues. Survival plot **4A** is for OS and **4B** for PFS

**S4C-D:** Kaplan-Meier survival plots stratified by tumor tissue purity, between high and low DNA tissue purity. Survival plots are based on OS (**4C**) and PFS (**4D**)

## **Supplementary Figure 5: Association of AR score and NEPC score with overall survival (OS) in treatment naive cBioPortal tissue biospecimen**


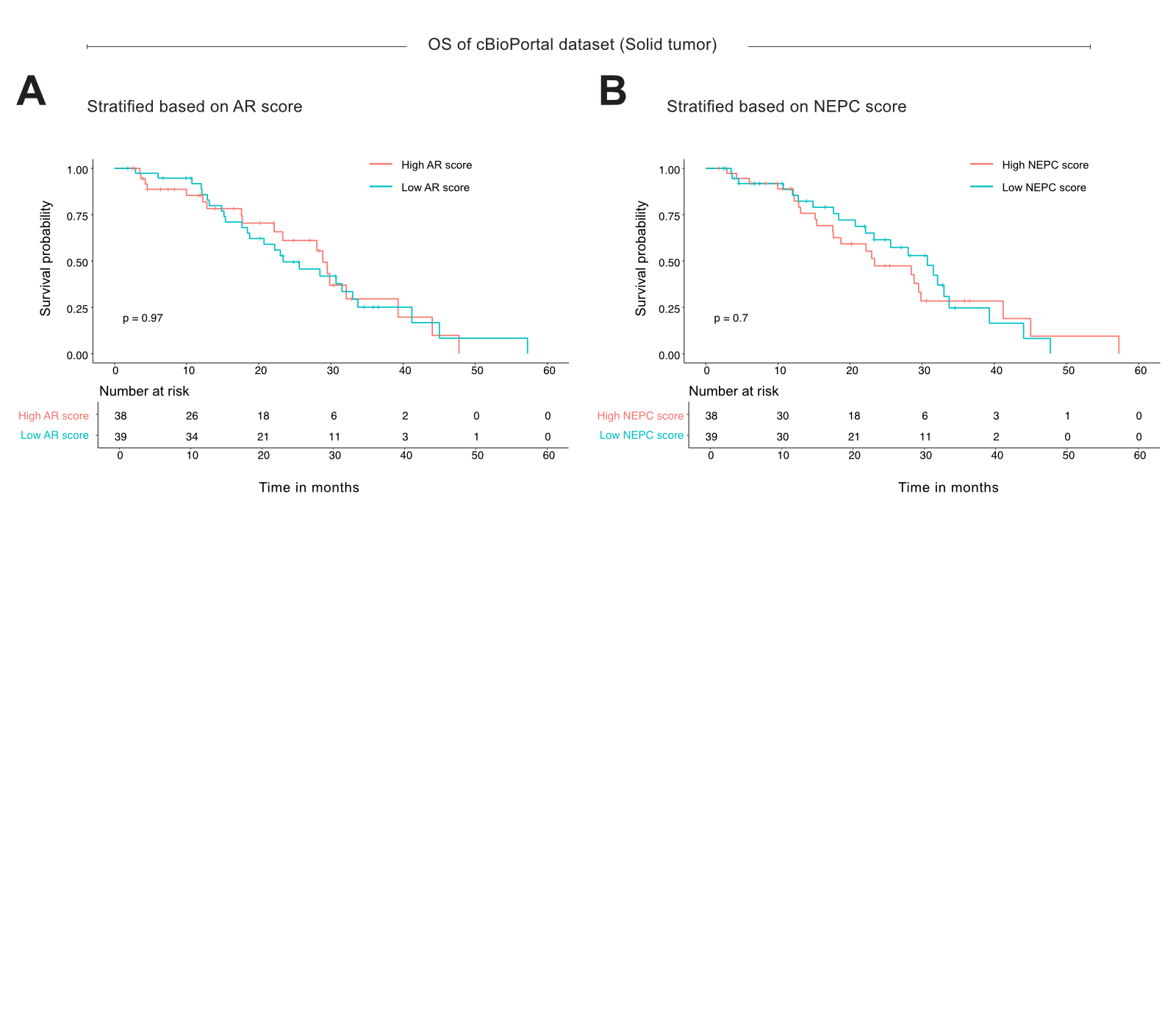


**Supplementary Figure 5: Association of AR score and NEPC score with overall survival (OS) in treatment naive cBioPortal tissue biospecimen**

**S5A-B**: Kaplan-Meier survival plots stratified by AR score (A) and NEPC score (B).
